# Convergence of Modality Invariance and Attention Selectivity in the Cortical Semantic Circuit

**DOI:** 10.1093/cercor/bhab125

**Published:** 2021-05-17

**Authors:** Tomoya Nakai, Hiroto Q Yamaguchi, Shinji Nishimoto

**Affiliations:** Center for Information and Neural Networks (CiNet), National Institute of Information and Communications Technology, Osaka 565-0871, Japan; Graduate School of Frontier Biosciences, Osaka University, Osaka 565-0871, Japan; Center for Information and Neural Networks (CiNet), National Institute of Information and Communications Technology, Osaka 565-0871, Japan; Graduate School of Frontier Biosciences, Osaka University, Osaka 565-0871, Japan; Center for Information and Neural Networks (CiNet), National Institute of Information and Communications Technology, Osaka 565-0871, Japan; Graduate School of Frontier Biosciences, Osaka University, Osaka 565-0871, Japan; Graduate School of Medicine, Osaka University, Osaka 565-0871, Japan

**Keywords:** attention selectivity, encoding model, functional magnetic resonance imaging, language, modality invariance

## Abstract

The human linguistic system is characterized by modality invariance and attention selectivity. Previous studies have examined these properties independently and reported perisylvian region involvement for both; however, their relationship and the linguistic information they harbor remain unknown. Participants were assessed by functional magnetic resonance imaging, while spoken narratives (auditory) and written texts (visual) were presented, either separately or simultaneously. Participants were asked to attend to one stimulus when both were presented. We extracted phonemic and semantic features from these auditory and visual modalities, to train multiple, voxel-wise encoding models. Cross-modal examinations of the trained models revealed that perisylvian regions were associated with modality-invariant semantic representations. Attentional selectivity was quantified by examining the modeling performance for attended and unattended conditions. We have determined that perisylvian regions exhibited attention selectivity. Both modality invariance and attention selectivity are both prominent in models that use semantic but not phonemic features. Modality invariance was significantly correlated with attention selectivity in some brain regions; however, we also identified cortical regions associated with only modality invariance or only attention selectivity. Thus, paying selective attention to a specific sensory input modality may regulate the semantic information that is partly processed in brain networks that are shared across modalities.

## Introduction

Modality invariance (MI) and attention selectivity (AS) are two properties that are characteristic of language communication. We understand linguistic contents, regardless of their presentation in text or speech (MI). When we are exposed to different linguistic stimuli simultaneously, however, attending to auditory information often prevents the understanding of information presented visually (AS).

Previous studies have reported modality-invariant brain activity, associated with single-word processing (Booth et al. 2002; Marinkovic et al. 2003), sentence comprehension (Carpentier et al. 2001; Jobard et al. 2007), and story comprehension (Deniz et al. 2019; Regev et al. 2013; Nguyen et al. 2019). In particular, Deniz et al. (2019) quantitatively estimated common semantic information across visual and auditory modalities, even after excluding the effects of other linguistic and sensory features. Modality-invariant linguistic information is likely represented in the perisylvian, “higher-order” brain regions, including the inferior frontal, superior temporal, and parietal regions (Regev et al. 2013).

In contrast, other studies have reported that selective attention can improve the comprehension of sentences in the attended modality and induce changes in cortical activity patterns (Moisala et al. 2015; Regev et al. 2019; Wang and He 2014). Regev et al. (2019) showed that selective attention can enhance the linguistic information flow of the attended modality, from early sensory areas along the processing hierarchy, converging in the perisylvian, “higher-order” brain regions.

**Figure 1:**
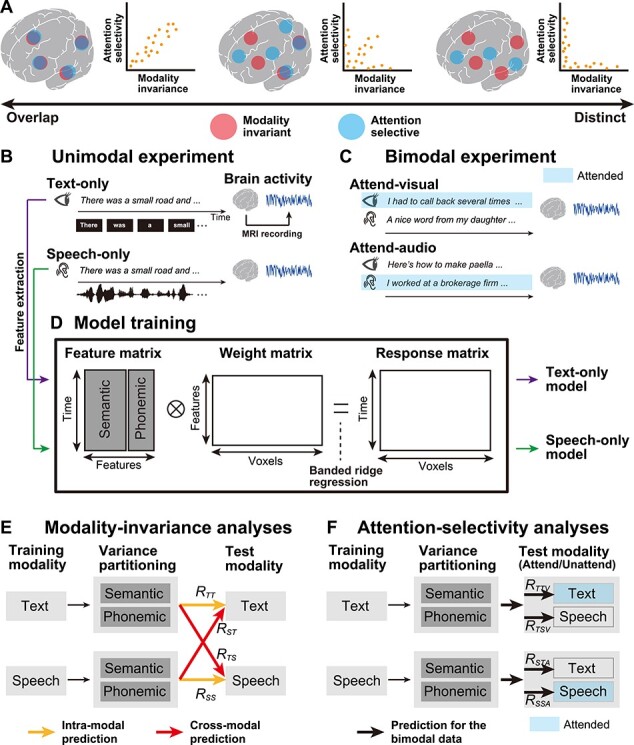
Schematic image of the experimental paradigm and the encoding modeling. (*A*) Three possible hypotheses are possible regarding the overlap between brain regions associated with modality-invariant linguistic representations (red) and those modulated by selective attention (blue). Brain regions showing these higher-order properties could be overlapping (left), independent (right), or partially overlapping (center). (*B*) Experimental design. During the unimodal experiment (left panel), participants passively listened to auditory stimuli, in the Speech-only condition, or read a written text, in the Text-only condition. Brain activity was measured using fMRI. The original Japanese stimuli were translated into English for the purpose of intelligibility. (*C*) During the bimodal experiment (right panel), visual and auditory stimuli were presented to participants, simultaneously. The participants selectively attended to the visual (Attend-visual condition) or auditory (Attend-audio condition) modality. The stimuli in the attended modality are highlighted in blue. (*D*) Semantic and phonemic features were extracted from the text and speech stimuli used during the unimodal experiment, and encoding models were separately trained, using the brain activity of the training dataset (text-only and speech-only models). For model training, using a concatenated matrix of semantic and phonemic features, we used a banded ridge regression (see Materials and Methods). (*E*) For MI analyses, trained unimodal models were used to predict brain activity in the test dataset from the unimodal experiments, in both intramodal (yellow arrows) and cross-modal (red arrows) manners. The prediction accuracy notations are described with each arrow (see Table 1 for a description of all notations). Semantic and phonemic components were separated using variance partitioning analysis (see Materials and Methods). (*F*) For AS analyses, the trained unimodal models were used to predict brain activity during the bimodal experiment, and semantic and phonemic features were extracted from both attended and unattended modalities. Stimuli in the attended modality are highlighted in blue.

Although the processing hierarchy of linguistic information has been suggested, in terms of both MI and AS, independently, how these two “higher-order” areas are related to each other is yet to be determined (Fig. 1*A*). The first hypothesis is that these areas overlap, forming a unified area that represents modality-invariant and attention-selective information (Fig. 1*A*, left). The second hypothesis is that functionally distinct areas operate independently (Fig. 1*A*, right). The third hypothesis exists between these two extremes: some areas represent both MI and AS, whereas other areas exclusively represent one or the other (Fig. 1*A*, center). Which types of linguistic information (semantic or phonemic) contribute to these properties also remains unknown.

To address these issues, 7-h, functional magnetic resonance imaging (fMRI) experiments were conducted, during which unimodal and bimodal language stimuli were presented. During the unimodal experiments (Fig. 1*B*), six participants either listened to spoken narratives (Speech-only condition) or read transcribed narratives (Text-only condition). Meanwhile, during the bimodal experiment (Fig. 1*C*), both speech and text were presented simultaneously, and the participants were asked to attend to either the speech or the text (Attend-audio or Attend-visual conditions, respectively). Data from the unimodal experiment were used to evaluate MI, whereas the data from the bimodal experiment were used to evaluate AS.

In order to evaluate the brain representations of multiple linguistic features, quantitatively, we used voxel-wise encoding models (Naselaris et al. 2011) (Fig. 1*D*). By using this approach, brain activity can be modeled by a combination of features that are extracted from the presented stimuli. Researchers have adopted this approach to comprehensively examine semantic representations (de Heer et al. 2017; Deniz et al. 2019; Huth et al. 2016), visual object category representations (Çukur et al. 2013a; Çukur et al. 2016; Çelik et al. 2019; Huth et al. 2012), and how attention modulates representations (Çukur et al. 2013b; Shahdloo et al. 2020). Using this modeling approach, under cross-modal and multiple-attention conditions, we elucidated a quantitative relationship between modality-invariant representations (Fig. 1*E*) and attentional modulation (Fig. 1*F*), in a feature-specific manner.

## Materials and Methods

### Participants

Six healthy participants (referred to as ID01–ID06; ages 22–29; all native Japanese; two females), with normal vision and hearing, participated in the fMRI experiments. Participants were all right-handed, as measured using the Edinburgh inventory (Oldfield 1971) (laterality quotients, 62.5–100). Informed consent was obtained from all participants, prior to their participation in the study. This experiment has received approval from the Ethics and Safety Committee of the National Institute of Information and Communications Technology, Osaka, Japan.

### Stimuli and Tasks

We selected 20 narrative stories from the Corpus of Spontaneous Japanese (Maekawa, 2003), of which 14 narratives were used during the training runs, for both Text-only and Speech-only conditions (total of 28 runs). One narrative was used only in the test run of the Text-only condition, one narrative was used only in the test run of the Speech-only condition, two narratives were used only in the test run of the Attend-visual condition (i.e., simultaneously presented in a single run), and two narratives were used only in the test run of the Attend-audio condition (i.e., simultaneously presented in a single run). All test runs were conducted twice (total of eight runs). We used different narratives during each test runs, in order to avoid adaptations to the redundant presentation of the same content.

All narratives were originally recorded in the auditory modality. Sound signals were controlled by their root mean square and were only used in the Speech-only, Attend-visual, and Attend-audio conditions. Visual stimuli used for the Text-only, Attend-visual, and Attend-audio conditions were generated by presenting each spoken segment on the center of the screen. The onset of each visual segment has matched the onset of the corresponding segment in the spoken narrative. The average duration of the spoken narratives (mean ± standard deviation [SD]) was 673 ± 70 s.

During the Speech-only condition, participants were asked to fixate on a fixation cross-presented on the center of the screen and listened to spoken narratives, through MRI-compatible ear tips. Meanwhile, during the Text-only condition, participants read the transcribed narratives, which were displayed on the center of the screen, using a Rapid Serial Visual Presentation method (Forster 1970). During the Attend-audio condition, participants listened to the spoken narratives, through MRI-compatible ear tips, and were instructed to ignore the text that was displayed simultaneously. Participants were asked not to close their eyes and were further instructed to fixate on the center of the screen. During the Attend-visual condition, participants were instructed to read the transcribed narratives displayed on the center of the screen, while ignoring the simultaneously presented spoken narratives.

At the beginning of each run, 10 s of dummy scans were acquired, during which the fixation cross was displayed, and these dummy scans were later omitted from the final analysis to reduce noise. We also obtained 10 s of scans at the end of each run, during which the fixation cross was displayed, and these were included in the analyses. In total, 36 fMRI runs were performed for each participant. Among these, 28 runs were used for model training (14 each, under the Speech-only and Text-only conditions), whereas 8 runs were performed for model testing (2 each, under the Text-only, Speech-only, Attend-visual, and Attend-audio conditions). For each participant, the experiments were executed over the course of 7 days, with 4–6 runs performed each day.

Participants were informed prior to the fMRI scan that, after each run, they would be asked to answer 10 questions relevant to the stimulus on which they were instructed to concentrate (the attended stimulus). However, the actual questionnaire that was administered after the fMRI scans included 10 questions that were relevant to both the attended and unattended stimuli. This surprise was intended so that participants would concentrate on understanding the instructed modality while ignoring the distractive one.

### MRI Data Acquisition

This experiment was conducted on a 3.0T MRI scanner (MAGNETON Prisma; Siemens, Erlangen, Germany), with a 64-channel head coil. We scanned 72 2.0-mm-thick interleaved, axial slices, without a gap, using a T2-weighted, gradient-echo, multiband, echo-planar imaging sequence (Moeller et al. 2010) (repetition time [TR] = 1000 ms, echo time [TE] = 30 ms, flip angle [FA] = 62°, field of view [FOV] = 192 × 192 mm^2^, voxel size = 2 × 2 × 2 mm^3^, multiband factor = 6). The number of volume collected was determined to be different for each run, depending on the stimuli length, of (mean ± SD) 693 ± 70 s (including the 10 s of initial dummy scans and the 10 s of fixation scans at the end of each run). For anatomical reference, high-resolution T1-weighted images of the whole brain were also acquired from all participants, using a magnetization-prepared rapid acquisition gradient-echo sequence (MPRAGE, TR = 2530 ms, TE = 3.26 ms, FA = 9°, FOV = 256 × 256 mm^2^, voxel size = 1 × 1 × 1 mm^3^).

### Semantic Features

To quantitatively evaluate the brain representations of the presented semantic information, in a data-driven manner, we extracted the semantic features from each narrative stimulus, using Wikipedia2Vec (Yamada et al. 2018; https://wikipedia2vec.github.io/wikipedia2vec/). Wikipedia2Vec has been used to embed words and entities into distributed representations, based on the skip-gram model (Mikolov et al. 2013). The Wikipedia2Vec model is considered to be an extension of the conventional Word2Vec model, which we used in our previous study (Nishida and Nishimoto 2018). The Word2Vec model is trained solely on contextual words around a target word and has difficulty in dealing with entities (e.g., New York and Julius Caesar). In contrast, the Wikipedia2Vec model is trained on both contextual words and entity link information. All transcribed narrative segments were further segmented into words and were morphologically parsed, using MeCab (https://taku910.github.io/mecab/). Individual word segments were projected into the 300-dimensional space (i.e., word vectors with 300 elements) and were later assigned to the mean time point between the onset and offset of target segments, with 40 Hz. The dimension size of the word vectors was set to the default value of 300. Time points without any word vector assignments were defined as 0. The resultant concatenated vectors were downsampled to 1 Hz.

To confirm that the narratives used in the current study were sufficiently covered in the Wikipedia2Vec semantic space, we calculated the ratio of words contained in the current narratives to words contained in the training dataset of the Wikipedia2Vec model. The resultant ratio was 3582/3902 = 0.918, which indicated that the current narratives were adequately covered in the Wikipedia2Vec semantic space.

### Phonemic Feature

To compare the predictability of brain activity, based on semantic features, with that of other non-semantic linguistic features, we also extracted phonemic features from each narrative stimulus. By using the Julius speech recognition software (Lee et al. 2001), an onset of each phoneme included in each spoken narrative was extracted. Each phoneme was then temporally aligned, based on the estimated onset. In total, 39 phonemes were extracted using the phoneme alignment procedure. Each phoneme was assigned to the mean time point between its onset and offset, and the number of phonemes presented each second was used as a phonemic feature. Based on the literature on phonological coding (Leinenger 2014), we assumed that phonological processing is related not only to the listening of narrative stories but also to the reading of transcribed narratives (i.e., the Text-only condition), and phonemic features are assigned to the text stimuli according to their narrative counterpart.

### Letter Feature

To model the orthographical components of the Text-only condition, we extracted the number of letters presented each second as a letter feature.

### fMRI Data Preprocessing

Motion corrections for each run were performed using the Statistical Parametric Mapping Toolbox (SPM8). All volumes were aligned using the first echo-planar imaging result for each participant. Low-frequency drift was removed, using a median filter, with a 120-s window. The response for each voxel was then normalized, by subtracting the mean response and scaling the response to the unit variance. We used FreeSurfer (Dale et al. 1999; Fischl et al. 1999) to identify cortical surfaces, based on anatomical data, and to register them against voxels for functional data. For each participant, voxels identified throughout the whole brain were used in the analysis.

### Encoding Model Fitting

The cortical activity measured in each voxel was fitted with a finite impulse response (FIR) model that captured slow hemodynamic responses and its coupling with neural activity (Nishimoto et al. 2011; Kay et al. 2008). Although many fMRI studies use canonical hemodynamic response function, this function assumes that the hemodynamic response function has the same shape across all cortical voxels. This may lead to an inaccurate modeling of brain activity because there is variation in hemodynamic responses across different cortical regions (Handwerker et al. 2004). Using the FIR model, we estimated voxel-specific hemodynamic response functions (Kay et al. 2008). Potential overfitting of the FIR model was avoided by using regularization. In order to examine how the different linguistic features were associated with cortical activity patterns, we modeled brain activity using two linguistic features (phonemic and semantic). A semantic feature matrix, *F*_1_ [*T* × 6 *N*_1_], was modeled by concatenating sets of [*T* × *N*_1_] semantic feature matrices, with six temporal delays of 2–7 s each (*T* = no. of samples; *N*_1_ = no. of features). Similarly, the phonemic feature matrix, *F*_2_ [*T* × 6 *N*_2_], was modeled using concatenating sets of [*T* × *N*_2_], using phonemic feature matrices, with six temporal delays. A cortical response, *X* [*T* × *V*], was then modeled by the concatenated feature matrices of *F*_1_ and *F*_2_, multiplied by the concatenated weight matrices, *W*_1_ [6N_1_ × *V*] and *W*_2_ [6*N*_2_ × *V*] (*V* = no. of voxels):

$$ X=\left[{F}_1\ {F}_2\right]\left[\genfrac{}{}{0pt}{}{W_1}{W_2}\right]+\varepsilon $$

where $\varepsilon$ is isotropic gaussian noise. In analyzing the predictive performance of the two linguistic models exclusively, we used banded ridge regression on the training dataset to obtain the weight matrices, *W*_1_ and *W*_2_ (Nunez-Elizalde et al. 2019). Specifically, weight matrices were estimated by solving the following equation, with regularization parameters ${\alpha}_1$ and ${\alpha}_2$:

$$ \left[\genfrac{}{}{0pt}{}{{\hat{W}}_1}{{\hat{W}}_2}\right]=\underset{W_1,{W}_2}{\mathrm{argmin}}\left[{\left\Vert X-{F}_1{W}_1-{F}_2{W}_2\right\Vert}_2^2+{\alpha}_1{\left\Vert{W}_1\right\Vert}_2^2+{\alpha}_2{\left\Vert{W}_2\right\Vert}_2^2\right] $$

The solution to this equation is as follows:

$$ \left[\genfrac{}{}{0pt}{}{{\hat{W}}_1}{{\hat{W}}_2}\right]=\left(\left[\begin{array}{@{}cc}{F}_1^T{F}_1& {F}_1^T{F}_2\\{}{F}_2^T{F}_1& {F}_2^T{F}_2\end{array}\kern-4pt\right]+\left[\begin{array}{@{}cc}{\alpha}_1{I}_1& 0\\{}0& {\alpha}_2{I}_2\end{array}\kern-4pt\right]\right)\left[\genfrac{}{}{0pt}{}{F_1^T}{F_2^T}\right]X $$

where *I*_1_ and *I*_2_ are identity matrices of the sizes [6*N*_1_ × 6 *N*_1_] and [6*N*_2_ × 6 *N*_2_], respectively. The training dataset consisted of 9815 samples, under both Speech-only and Text-only conditions. An optimal regularization parameter was assessed in each voxel using 10-fold cross-validation.

The test dataset consisted of 619 Speech-only samples, 617 Text-only samples, 613 Attend-audio samples, and 623 Attend-visual samples. Differences in sample sizes in the test dataset can be attributed to the various durations of the naturalistic narrative story stimuli. Two repetitions of the test dataset were averaged to increase the signal-to-noise ratio.

### Encoding Model Fitting Using Visual and Auditory Regressors

To exclude the effect of sensory information, we constructed additional encoding models using visual and auditory regressors. For the visual regressor, we used a motion energy model (Nishimoto et al. 2011). First, movie frames and pictures were spatially downsampled to 96 × 96 pixels. The RGB pixel values were then converted into the Commission International de l’Eclairage LAB color space, and the color information was subsequently discarded. The luminance (L*) pattern was passed through a bank of three-dimensional spatiotemporal Gabor wavelet filters. The outputs of the two filters with orthogonal phases (quadrature pairs) were squared and summed to yield the local ME. ME was compressed with a log-transformation and temporally downsampled to 1 Hz. Filters were tuned to six spatial frequencies (0.75, 1.5, 3.0, 6.0, 12.0, 24.0 cycles per image) and three temporal frequencies (1.0, 2.0, 4.0 Hz), without directional parameters. Filters were positioned on a square grid that covered the screen. The adjacent filters were separated by 4.0 SD of their spatial Gaussian envelopes. The original ME features (1920) were reduced to 300 dimensions using principal component analysis (PCA).

For the auditory regressor, we used a modulation transfer function (MTF) model (Nakai et al. 2021). A sound cochleogram was generated using a bank of 128 overlapping bandpass filters that ranged from 20 to 10 000 Hz. The window size was set to 25 ms and the hop size was set to 10 ms. The filter output was averaged across 1 s (TR). We further extracted the features from the MTF model, which we have provided in a public repository. For each cochleogram, we calculated a convolution with modulation-selective filters. The outputs of the two filters with orthogonal phases (quadrature pairs) were squared and summed to yield the local modulation energy. Modulation energy was then log-transformed, averaged across 1 s, and further averaged within each of the 20 nonoverlapping frequency ranges that were logarithmically spaced along the frequency axis. The filter outputs of the upward and downward sweep directions were also averaged. Modulation-selective filters were tuned to 10 spectral modulation scales (0.35, 0.50, 0.71, 1.00, 1.41, 2.00, 2.83, 4.00, 5.66, and 8.00 cycles per octave) and 10 temporal modulation rates (2.8, 4.0, 5.7, 8.0, 11.3, 16.0, 22.6, 32.0, 45.3, and 64.0 Hz). The original MTF features (2000) were reduced to 300 dimensions using PCA.

For the model training of the Text-only condition, the visual regressor was concatenated with the nontarget features in the banded-ridge regression (if the target features were semantic features, the regressor was concatenated with the phonemic features). The effect of visual information was then excluded through variance partitioning analysis. This analysis was performed for each of the semantic and phonemic features as target features. For the model training of the Speech-only condition, the auditory regressor was used in the same manner.

### Variance Partitioning Analysis

To assess the predictive performances of semantic and phonemic features separately, we performed a variance partitioning analysis (de Heer et al. 2017; Lescroart et al. 2015). Predicted signals were estimated for each of the two separate models and the concatenated model, as follows:

$$ {\displaystyle \begin{array}{c}{\hat{X}}_1={F}_1{W}_1\\[6pt] {}{\hat{X}}_2={F}_2{W}_2\\[6pt] {}{\hat{X}}_3=\left[{F}_1\ {F}_2\right]\left[\kern-4pt\begin{array}{c}{W}_1\\{}{W}_2\end{array}\kern-4pt\right]\end{array}} $$

Coefficients of determination were estimated for each of the two separate models and the concatenated model, as follows:

$$ {V}_i^2=1-\frac{\sum{\left({\hat{X}}_i-X\right)}^2}{\sum{\left(X-\overline{X}\right)}^2},\left(i=1,2,3\right) $$

where $X$ and $\overline{X}$ represent cortical response and mean response (across time) in the test dataset, respectively. Prediction accuracies for every single model (*R*_1_ and *R*_2_, for the semantic and phonemic features, respectively) were obtained by subtracting the coefficient of determinant calculated for a targeted single model from that calculated for the concatenated model, as follows:

$$ {\displaystyle \begin{array}{c}{R}_1^2={V}_3^2-{V}_2^2\\[6pt] {}{R}_2^2={V}_3^2-{V}_1^2\end{array}} $$

To make the predicted performance comparable with those reported by previous studies (de Heer et al. 2017; Deniz et al. 2019; Huth et al. 2016, 2012), the square root was calculated. To obtain a null distribution of the prediction accuracy, we calculated *R*_1_ and *R*_2_ values for all cortical voxels, based on the originally predicted responses and a random phase permutation of the actual responses from the test dataset. The resulting *P*-values (one-sided) were corrected for multiple comparisons using the false discovery rate (FDR) procedure (Benjamini and Hochberg 1995).

### Modality Invariance

To quantify how the unimodal models explained brain activity in each voxel, regardless of the presentation modality, we defined a MI value. Previous studies have quantified MI using a model weight correlation (Deniz et al. 2019) or intersubject correlation of brain activity (Nguyen et al. 2019). To quantify MI based on prediction accuracy, we used the geometric mean of prediction accuracy instead of weight correlation. This can be justified by the fact that models with similar weight values have similar predictive performance. MI consisted of two components: *D*_T_ and *D*_S_. *D*_T_ has been defined as a degree of predictability for the Text-only test dataset, regardless of the training modality:

$$ {D}_{\mathrm{T}}=\sqrt{R_{\mathrm{T}\mathrm{T}}\cdot{R}_{\mathrm{ST}}} $$

where ${R}_{\mathrm{TT}}$ and ${R}_{\mathrm{ST}}$ are the intramodal prediction accuracy for the text-only model and the cross-modal prediction accuracy calculated for the speech-only model when applied to the test dataset for the Text-only condition, respectively (see Table 1 for all notations of prediction accuracies). Note that the R_**_ values correspond to the *R*_1_ or *R*_2_ values described in the previous subsection, depending on the linguistic features used (i.e., semantic or phonemic). Similarly, *D*_S_ is defined as the degree of predictability calculated for the Speech-only test dataset, regardless of the training modality:

$$ {D}_{\mathrm{S}}=\sqrt{R_{\mathrm{TS}}\cdot{R}_{\mathrm{S}\mathrm{S}}} $$

where ${R}_{\mathrm{SS}}$ and ${R}_{\mathrm{TS}}$ are the intra-modal prediction accuracy by the speech-only model and the cross-modal prediction accuracy calculated for the text-only model when applied to the test dataset for the Speech-only condition, respectively. For all voxels showing negative prediction accuracies, the prediction accuracy was set to 0 to avoid obtaining imaginary values. MI was then calculated for each voxel as a geometric mean between ${D}_{\mathrm{S}}$ and ${D}_{\mathrm{T}}$, as follows:

$$ \mathrm{MI}=\sqrt{D_{\mathrm{S}}\cdot{D}_{\mathrm{T}}} $$

**Table 1:** Notation of prediction accuracy for all combinations of conditions, for both the training and test datasets

MI analyses (unimodal experiments)				
Training condition	Test condition and feature modality			
	Text-only condition (Features from the text)		Speech-only condition (Features from speech)	
Text-only condition	${R}_{\mathrm{TT}}$		${R}_{\mathrm{TS}}$	
Speech-only condition	${R}_{\mathrm{ST}}$		${R}_{\mathrm{SS}}$	
AS analyses (bimodal experiments)				
	Attend-visual condition		Attend-audio condition	
	Features from the text (attended)	Features from speech (unattended)	Features from the text (unattended)	Features from speech (attended)
Text-only condition	${R}_{\mathrm{TTV}}$	${R}_{\mathrm{TSV}}$		
Speech-only condition			${R}_{\mathrm{STA}}$	${R}_{\mathrm{SSA}}$

MI value ranges from 0 to 1. A high MI value indicates that the target linguistic features are represented in a modality-independent manner, where MI = 0 indicates that the target voxel does not have a shared linguistic representation of text and speech. The significance of each MI value was assessed using a phase permutation test (one-sided), corrected for multiple comparisons using the FDR procedure (Benjamini and Hochberg 1995).

As an additional analysis, we also calculated *D*_T_, *D*_S_, and $\mathrm{MI}$ using the arithmetic mean (denoted as ${D_{\mathrm{T}}}^{\mathrm{Arith}}$, ${D_{\mathrm{S}}}^{\mathrm{Arith}}$, and ${\mathrm{MI}}^{\mathrm{Arith}}$):

$$ {\displaystyle \begin{array}{c}{D_{\mathrm{T}}}^{\mathrm{Arith}}=\left({R}_{\mathrm{T}\mathrm{T}}+{R}_{\mathrm{S}\mathrm{T}}\right)/2\\[3pt] {}{D_{\mathrm{S}}}^{\mathrm{Arith}}=\left({R}_{\mathrm{T}\mathrm{S}}+{R}_{\mathrm{S}\mathrm{S}}\right)/2\\[3pt] {}{\mathrm{MI}}^{\mathrm{Arith}}=\left({D_{\mathrm{S}}}^{\mathrm{Arith}}+{D_{\mathrm{T}}}^{\mathrm{Arith}}\right)/2\end{array}} $$

The significance of ${\mathrm{MI}}^{\mathrm{Arith}}$ was assessed similarly to the original $\mathrm{MI}$.

### Modality Specificity

To quantify how the unimodal models explained brain activity that was specific for a single modality, we defined modality specificity, which was calculated in each voxel for each modality (MS_T_ for the Text-only condition and MS_S_ for the Speech-only condition) as the difference between the intramodal and cross-modal prediction accuracies:

$$ {\displaystyle \begin{array}{c}{\mathrm{MS}}_{\mathrm{T}}={R}_{\mathrm{T}\mathrm{T}}\hbox{-} {R}_{\mathrm{S}\mathrm{T}}\\[3pt] {}{\mathrm{MS}}_{\mathrm{S}}={R}_{\mathrm{S}\mathrm{S}}\hbox{-} {R}_{\mathrm{T}\mathrm{S}}\end{array}} $$

MS value ranges from −1 to 1. A high MS value indicates that the target linguistic features are represented specifically according to the target modality, where negative MS indicates that the target voxel does not have a modality-specific representation. Significance and FDR corrections for multiple comparisons were calculated as described for the MI values.

### Attention Selectivity

To quantify how each cortical voxel was affected by selective attention, we defined an AS value. Based on a previous study that contrasted brain activity between attended and unattended conditions (Regev et al. 2019), the effect of selective attention was measured by the difference in prediction accuracy between the attended and unattended conditions. AS consisted of two components, ${A}_V$ and ${A}_A$, which indicated the augmentation of prediction accuracies according to the application of selective attention to the visual and auditory modalities, respectively. To calculate ${A}_V$, the text-only model was tested on the test dataset acquired under the Attend-visual condition. The prediction accuracies contrasted the features from the visual (attended) and the auditory (unattended) modalities, as follows:

$$ {A}_{\mathrm{V}}={R}_{\mathrm{TTV}}-{R}_{\mathrm{TSV}} $$

where ${R}_{\mathrm{TTV}}$ and ${R}_{\mathrm{TSV}}$ represent the prediction accuracies calculated based on the features from the visual (attended) and auditory (unattended) modalities, respectively (see Table 1 for all notations of prediction accuracies). Similarly, to calculate ${A}_{\mathrm{A}}$, the speech-only model was tested on the test dataset acquired under the Attend-audio condition. The prediction accuracies contrasted the features from the auditory (attended) and visual (unattended) modalities, as follows:

$$ {A}_{\mathrm{A}}={R}_{\mathrm{SSA}}-{R}_{\mathrm{STA}} $$

where ${R}_{\mathrm{SSA}}$ and ${R}_{\mathrm{STA}}$ are the prediction accuracies calculated based on the features from the auditory (attended) and visual (unattended) modalities, respectively. Voxels showing negative ${A}_{\mathrm{V}}$ and ${A}_{\mathrm{A}}$ values were set to 0. AS was calculated as the geometric mean of ${A}_{\mathrm{V}}$ and ${A}_{\mathrm{A}}$, as follows:

$$ \mathrm{AS}=\sqrt{A_{\mathrm{V}}\cdot{A}_{\mathrm{A}}} $$

AS value ranges from 0 to 1. AS is high when the features extracted from the attended modality always predict brain activity more accurately than those of the unattended modality, where AS = 0 indicates that the linguistic representation of the target voxel is not affected by the selective attention. The calculation of statistical significance and FDR corrections for multiple comparisons were performed as described for the MI values.

As an additional analysis, we also calculated $\mathrm{AS}$ using the arithmetic mean (denoted as ${\mathrm{AS}}^{\mathrm{Arith}}$):

$$ {\mathrm{A}\mathrm{S}}^{\mathrm{A}\mathrm{rith}}=\left({A}_{\mathrm{V}}+{A}_{\mathrm{A}}\right)/2 $$

The significance of ${\mathrm{AS}}^{\mathrm{Arith}}$ was assessed similarly to the original $\mathrm{AS}$.

### Anatomical ROI Analysis of MI and AS

To quantify how different cortical regions display MI and AS, we calculated ratios between voxels with exclusively positive MI values (“MI-only voxels”), voxels with exclusively positive AS values (“AS-only voxels”), or voxels showing both positive MI and AS values (“shared voxels”) and voxels showing either positive MI or AS values across 148 anatomical regions of interest (ROIs), based on the Destrieux cortical atlas (Destrieux et al. 2010). To focus on the cortical regions associated with linguistic information, we selected ROIs that contained a relatively large number of voxels with positive MI or AS values (>30% and >100 voxels within the target ROI).

## Results

### Selective Attention Facilitated the Understanding of Semantic Content

To confirm that participants performed the selective attention task as instructed, we used a post-experimental questionnaire that tested whether participants understood the content of the attended stimuli. The average score of the post-experimental questionnaire was higher for the attended stimuli (mean ± SD, 90.8% ± 4.9%) than that of the unattended stimuli (50% ± 4.5%; chance level = 50%; *P* < 0.02 for all participants using chi-squared tests), which indicated that selective attention facilitated participants’ comprehension of the semantic content in the linguistic stimuli.

### The Semantic Encoding Model Predicted Brain Activity, Regardless of Modality

To confirm that the encoding models successfully captured brain activity during the unimodal experiments (Fig. 1*B*), we performed a series of intramodal encoding modeling tests and examined the modeling accuracy using a test dataset from the same modality as the training dataset (Fig. 1*E*, yellow). To quantifiably evaluate the predictability of brain activity, based on different linguistic information, we extracted both semantic and phonemic features from the narrative stimuli. We exclusively evaluated the effects of either semantic or phonemic features by combining banded ridge regression (Nunez-Elizalde et al. 2019) with variance partitioning analysis (de Heer et al. 2017; Lescroart et al. 2015) (see Materials and Methods for details).

**Figure 2:**
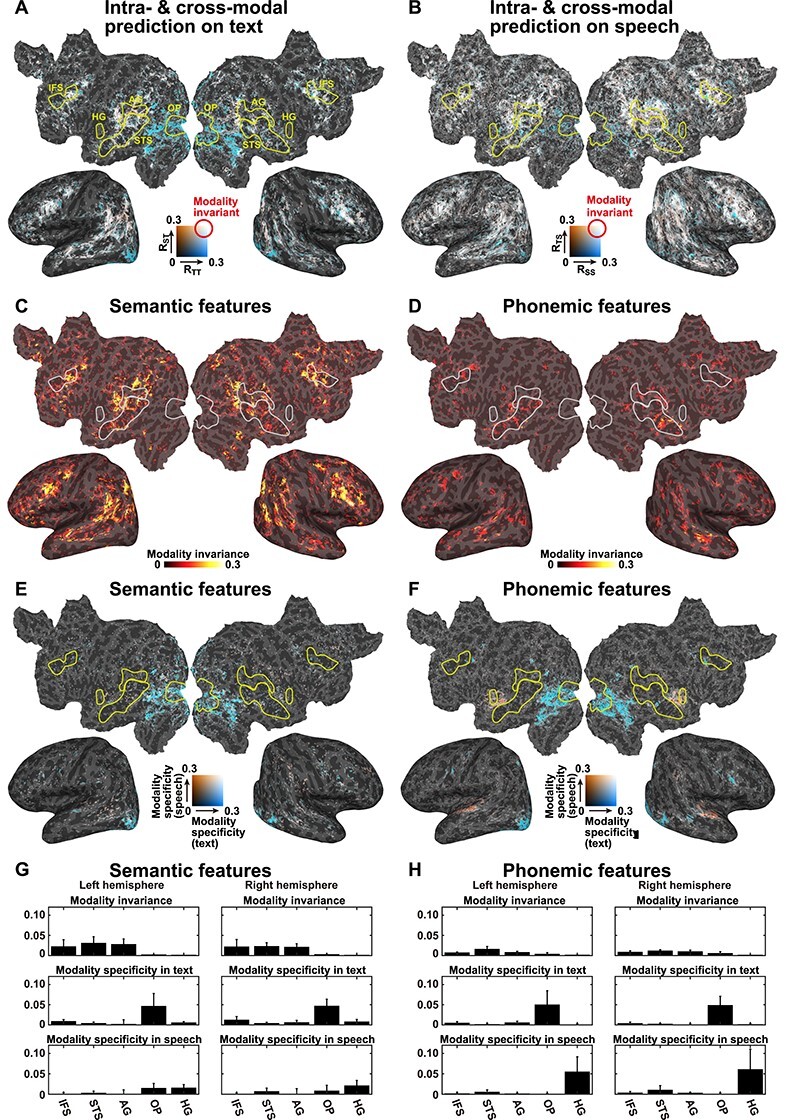
Effects of stimulus modality. (*A*) Comparison of the prediction accuracies of the unimodal models when applied to a Text-only test dataset using semantic features. The intramodal prediction accuracies of the text-only model (denoted as *R*_TT_, blue) and the cross-modal speech-only model (denoted as *R*_ST_, orange) are mapped onto the cortical surface of participant ID01. Regions in which activity was predicted, regardless of the stimulus modality, are shown in white. Only significant regions (*P* < 0.05, FDR corrected) are shown. Anatomical ROIs are marked by yellow lines. (*B*) Comparison between the prediction accuracies of the unimodal models for a Speech-only test dataset, using semantic features. Intramodal prediction accuracy, using the speech-only model (denoted as *R*_SS_, blue), and the cross-modal prediction accuracy, using the text-only model (denoted as *R*_TS_, orange), are mapped onto the cortical surface. MI was calculated using semantic features (*C*) or phonemic features (*D*) (see Supplementary Figs S2 and S3 for the other participants). Modality specificity for text (MS_T_, blue) and modality specificity for speech (MS_S_, red) were calculated using semantic features (*E*) or phonemic features (*F*) (see Supplementary Figs S4 and S5 for the other participants). Mean MI, MS_T_, and MS_S_ values were extracted from the five anatomical ROIs marked in (*A*), averaged across six participants, for both the left and right hemispheres, using semantic features (*G*) or phonemic features (*H*). Error bar, SD.

We first trained encoding models using the data from the Text-only condition (text-only model) and predicted brain activity using the text-only test dataset. Using semantic features, we found that the text-only model significantly predicted activity in the perisylvian regions, including the superior temporal, inferior frontal, and inferior parietal cortices (blue or white in Fig. 2*A*). Although we combined the phonemic features with the semantic features, prediction performance was not largely affected when letter features were used instead of phonemic features (Supplementary Fig. S1). Similarly, we trained encoding models using the data from the Speech-only condition (speech-only model) and predicted brain activity using the speech-only test dataset (blue or white in Fig. 2*B*). The speech-only model also significantly predicted activity in the perisylvian regions.

We next examined whether the unimodal models captured modality-invariant representations by performing cross-modal encoding modeling, during which we examined the modeling accuracy using a test dataset obtained from a different modality than the training dataset (Fig. 1*E*, red). A speech-only model was used to predict the brain activity with a text-only test dataset (*P* < 0.05, FDR corrected, red or white in Fig. 2*A*), which showed significant prediction accuracy in the perisylvian regions. Similarly, a text-only model was used to predict brain activity with a speech-only test dataset, which also displayed significant prediction accuracy in the perisylvian regions (red or white in Fig. 2*B*). The overlap between the intramodal and cross-modal prediction performances displayed a clear contrast between the cortical organization associated with modality-specific representation in the early sensory regions (i.e., the superior temporal and occipital regions) and that associated with the modality-invariant representation in the perisylvian regions.

To identify those regions that activate during modality-invariant representations of linguistic information, we further calculated the MI value, by combining the intramodal and cross-modal prediction accuracies, using semantic features. The MI values were determined to be significantly larger than 0 in the perisylvian regions (*P* < 0.05, FDR corrected, Fig. 2*C* and Supplementary Fig. S2), indicating that these regions are associated with modality-independent semantic representations. In contrast with semantic features, however, phonemic features were associated with small MI values across the cortex (Fig. 2*D* and Supplementary Fig. S3), indicating that modality-invariant representations of linguistic information are predominantly conveyed by semantic features.

To identify those regions associated with modality-specific representations, we calculated the modality specificity of text (MS_T_) and speech (MS_S_). Even though significantly higher MS_T_ values were observed in the visual cortex (*P* < 0.05, FDR corrected, blue in Fig. 2*E* and Supplementary Fig. S4), the MS_S_ values were significantly increased in the auditory cortex (*P* < 0.05, FDR corrected, red in Fig. 2*E* and Supplementary Fig. S4). Phonemic features were also significantly associated with MS_T_ values in the visual cortex and with MS_S_ values in the auditory cortex (Fig. 2*F* and Supplementary Fig. S5).

To evaluate the MI associated with each cortical region, we extracted MI values for five anatomical ROIs, averaged across all six participants (Fig. 2*G*,*H*). For the anatomical ROIs, we selected three perisylvian regions: the inferior frontal sulcus (IFS), the superior temporal sulcus (STS), and the angular gyrus (AG). Activity in these regions has frequently been reported in previous neuroimaging studies examining language (Price 2010). We also selected two sensory ROIs, at the occipital pole (OP) and Heschl’s gyrus (HG), which process early sensory components in the visual and auditory modalities, respectively. We found that the MI values for semantic features were larger in the three perisylvian regions than those in the sensory regions (Cohen’s *d* = 2.15 [left hemisphere], *d* = 2.32 [right hemisphere], calculated between the average of the three perisylvian regions and the average of the two sensory regions). In contrast, the MS_T_ values for semantic features in the three perisylvian regions were found to be smaller than those in the OP (*d* = 1.65 [left], *d* = 2.84 [right]), and the MS_S_ values for semantic features in the three perisylvian regions were smaller than those in the HG (*d* = 1.78 [left], *d* = 1.55 [right]). For the phonemic features, we also found that the MI values were larger in the perisylvian regions than those in the sensory regions (*d* = 2.62 [left], 2.21 [right]). In contrast, the MS_T_ values for semantic features in the three perisylvian regions were found to be smaller than those in the OP (*d* = 1.70 [left], *d* = 3.31 [right]), and the MS_S_ values for semantic features in the three perisylvian regions were smaller than those in the HG (*d* = 1.80 [left], *d* = 1.42 [right]).

**Figure 3:**
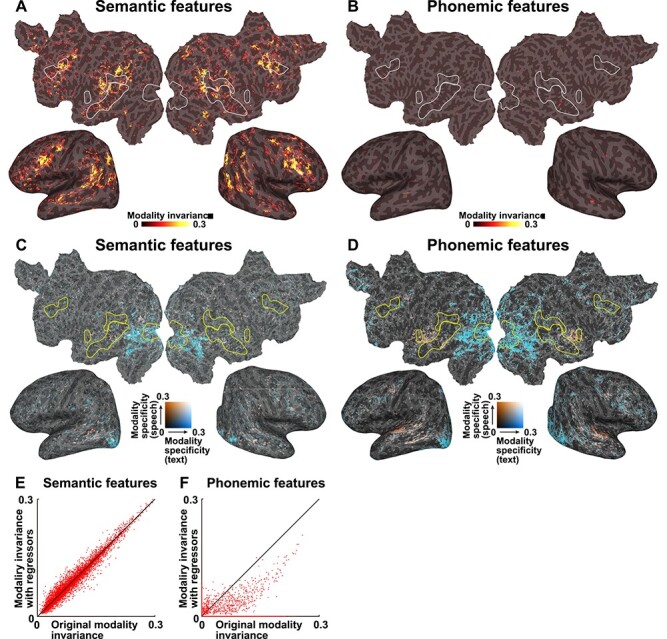
MI with sensory regressors. MI was calculated using semantic features (*A*) and phonemic features (*B*) and regressing out the sensory components and are mapped onto the cortical surface of participant ID01 (see Supplementary Figs S6 and S7 for the other participants). Only significant regions (*P* < 0.05, FDR corrected) are shown. Modality specificity for text (MS_T_, blue) and modality specificity for speech (MS_S_, red) were calculated using semantic features (*C*) and phonemic features (*D*) and regressing out sensory components (see Supplementary Figs S9 and S10 for the other participants). Scatter plots show the original MI, and MI with sensory regressors, for both semantic and phonemic features, plotted for participant ID01 (see Supplementary Fig. S8 for the other participants). Motion energy and MTF features were used as sensory regressors.

To exclude the effect of sensory information, we constructed additional encoding models by adding visual and auditory features as nuisance regressors (see Materials and Methods for details; Fig. 3 and Supplementary Figs S6–S10). The MI values of the semantic features were not largely affected by this analysis (Fig. 3*A*,*C*,*E* and Supplementary Figs S6, S8, S9). However, when we used the sensory regressors, the MI values of the phonemic features were reduced across the cortex (Fig. 3*B*,*D*,*F* and Supplementary Figs S7, S8, S10). These results also indicated that only semantic features are represented in the perisylvian regions in a modality-invariant manner, whereas modality-specific information for both the visual and auditory domains are represented in the primary sensory areas, regardless of linguistic features.

### Effect of Selective Attention on Model Prediction Performance

**Figure 4:**
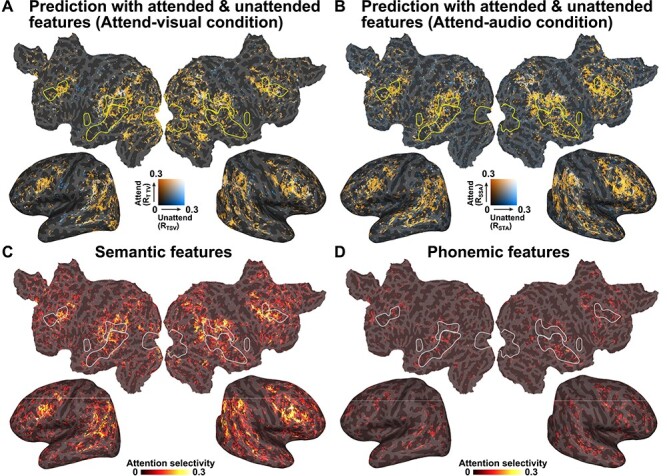
Effects of selective attention. (*A*) Prediction accuracies, using a text-only model on the Attend-visual condition dataset, mapped onto the cortical surface of participant ID01. Two prediction accuracies, associated with semantic features, were extracted from the attended modality (visual, orange) and the nonattended modality (audio, blue) and compared (*R*_TTV_ and *R*_TSV_, respectively). Only significant regions (*P* < 0.05, FDR corrected) are shown. (*B*) Comparison of the prediction accuracies using a speech-only model on the Attend-audio condition (*R*_SSA_ and *R*_STA_). AS was calculated by subtracting the prediction accuracy for the unattended condition from that for the attended condition and was mapped onto the cortical surface of participant ID01, using semantic features (*C*) or phonemic features (*D*) (see Supplementary Figs S11 and S12 for the other participants).

To examine whether selective attention affects the cortical representations of linguistic information, we conducted bimodal experiments, during which speech and text were simultaneously presented and participants were asked to selectively attend to only one of the two modalities (Fig. 1C). During the Attend-visual condition, we extracted semantic features from both the attended (visual) and unattended (auditory) modalities, which were presented simultaneously. The prediction accuracies were calculated by applying a text-only model, with features in each of the attended and unattended modalities (Fig. 1*F*, top). We found increased prediction accuracy across the cerebral cortex when using semantic features from the attended modality (Fig. 4*A*, orange), compared with those from the unattended modality (blue). Similarly, a speech-only model was tested using the data collected during the Attend-audio condition (Fig. 1*F*, bottom). We again found larger prediction accuracy across the cerebral cortex when using semantic features from the attended modality (Fig. 4*B*, orange), compared with those from the unattended modality (blue). Cross-modal prediction accuracies were not calculated during this procedure, and we evaluated MI and AS separately.

**Figure 5:**
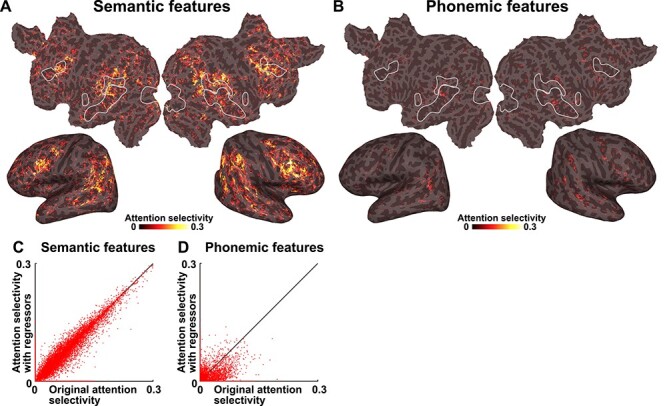
AS with sensory regressors. AS was calculated using semantic features (*A*) and phonemic features (*B*) and regressing out sensory components and is mapped onto the cortical surface of participant ID01 (see Supplementary Figs S13 and S14 for the other participants). Only significant regions (*P* < 0.05, FDR corrected) are shown. (*C*, *D*) Scatter plots show the original AS and AS with sensory regressors for both semantic and phonemic features, plotted for participant ID01 (see Supplementary Fig. S15 for the other participants). Motion energy and MTF features were used as sensory regressors.

To investigate which cortical regions were modulated by selective attention, we calculated AS by subtracting the prediction accuracy measured using unattended features from that calculated using attended features, within each modality (Fig. 4*A*,*B*). Larger AS values were identified in the inferior frontal, middle temporal, and inferior parietal regions when using semantic features (Fig. 4*C* and Supplementary Fig. S11). In contrast, we found that very small brain regions showed significant AS values when using phonemic features (Fig. 4*D* and Supplementary Fig. S12). In contrast to the MI values, the AS values were not largely affected by excluding sensory regressors (Fig. 5 and Supplementary Figs S13–S15).

### Select Brain Regions with Modality-Invariant Semantic Representations Are Affected by Selective Attention

**Figure 6:**
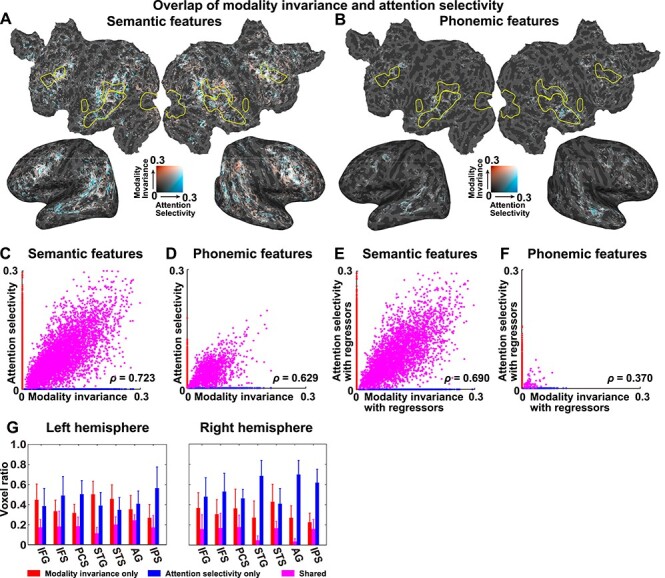
Partial overlap between the modality-invariant and attention-selective regions. MI (red) and AS (AS, blue) were mapped onto the cortical surface of participant ID01, using semantic features (*A*) and phonemic features (*B*). Only significant regions (*P* < 0.05, FDR corrected) are shown. A scatter plot is shown for MI and AS, using semantic features (*C*) and phonemic features (*D*) (see Supplementary Fig. S16 for the other participants). Data were extracted from the cortical voxels of participant ID01. Data with positive MI and zero AS are colored in red. Data with positive AS and zero MI are colored in blue. Data with positive MI and AS are colored in purple. For each plot, a Spearman’s correlation coefficient (*ρ*) is displayed. Scatter plots show the MI and AS values, using semantic features (*E*) and phonemic features (*F*), with sensory components regressed out (see Supplementary Fig. S17 for the other participants). Motion energy and MTF features were used as sensory regressors. (*G*) The ratios of voxels exclusively showing positive MI (red), those exclusively showing positive AS (blue), and those showing both MI and AS (purple) are plotted for seven anatomical ROIs in the left (left panel) and right hemispheres (right panel). IFG, inferior frontal gyrus. Error bar, SD.

An overlaid cortical map of the MI and AS values for semantic features (Fig. 6*A*,*B*) indicated that some voxels specifically represented MI (red), whereas other voxels specifically represented AS (blue). A scatter plot of the cortical voxels clearly revealed three types of voxels associated with semantic features (Fig. 6*C* and Supplementary Fig. S16), in which voxels associated with positive MI and 0 AS are colored in red (MI-only voxels; a mean ± SD voxel size across six participants, 5934 ± 2170), those associated with positive AS and 0 MI are colored in blue (AS-only voxels; 8727 ± 2311), and those associated with both positive MI and AS are colored in purple (shared voxels; 2186 ± 1550). Within the shared voxels, we found a positive correlation between AS and MI (Spearman’s correlation coefficients, *ρ* = 0.695 ± 0.025; Fig. 6*C*). In contrast, when using phonemic features, relatively few shared voxels were found to have significant values (Fig. 6*D*; MI-only voxels, 5462 ± 1233; AS-only voxels, 4423 ± 1451; shared voxels, 912 ± 449). However, we again found a positive correlation between AS and MI in these shared voxels (*ρ* = 0.610 ± 0.073).

The relationship between the MI and AS values was also examined using the encoding models that were constructed using sensory regressors. The distribution of the three types of voxels was not fully affected by regressing out the sensory components from the semantic features (Fig. 6*E*; MI-only voxels: 4914 ± 1882; AS-only voxels: 7282 ± 2481; shared voxels: 1659 ± 1158). For the phonemic features, we found few shared voxels after regressing out the sensory components (Fig. 6*F*; MI-only voxels: 1328 ± 388; AS-only voxels: 3639 ± 1462; shared voxels: 84 ± 19). We observed a significant positive correlation between the AS and MI values after regressing out the sensory components from the shared voxels (Fig. 6*E*,*F* and Supplementary Fig. S17; semantic features: *ρ* = 0.675 ± 0.042; phonemic features: *ρ* = 0.402 ± 0.100). These results indicated that the shared brain representation of MI and AS observed for the phonemic features (Fig. 6*D*) may have been caused by sensory information, whereas that observed for the semantic features was independent of sensory information.

In the scatter plots of the MI and AS values (Fig. 6*C*,*D*), we observed abrupt transitions from MI-only voxels to shared voxels and from shared voxels to AS-only voxels. Such abrupt transitions might have been caused by the definition of MI and AS based on the geometric mean. To clarify the underlying cause of such transitions, we calculated the MI and AS values based on the arithmetic mean. We found a similar distribution of MI values using the arithmetic mean as that of the original MI values (Spearman’s correlation coefficient for MI values calculated using the geometric and arithmetic means: *ρ* = 0.474 ± 0.080 for sematic features and *ρ* = 0.442 ± 0.036 for phonemic features; Supplementary Figs S18 and S19) and a similar distribution of AS values calculated using the arithmetic mean as that of the original AS values (Spearman’s correlation coefficient for AS values calculated using the geometric and arithmetic means: *ρ* = 0.549 ± 0.060 for sematic features and *ρ* = 0.388 ± 0.040 for phonemic features; Supplementary Figs S20 and S21), although their distributions were less localized within the perisylvian region and extended into the occipital cortex. The scatter plots of the MI and AS values demonstrated reduced abruptness in the transition from MI to shared voxels (Supplementary Fig. S22), which suggested that the abrupt transitions of the MI-only, shared, and AS-only voxels may have resulted from the current method of defining the MI and AS values.

To scrutinize the detailed cortical organization associated with MI and AS, we calculated the ratios between these three types of voxels and all voxels that display either positive MI or AS values across all of the anatomical ROIs when using semantic features (Fig. 6*G*). Because both MI and AS were more densely associated with semantic features than with phonemic features (Fig. 6*A*–*F*), we focused on semantic features in this analysis. Seven bilateral perisylvian ROIs (inferior frontal gyrus, IFS, precentral sulcus [PCS], superior temporal gyrus [STG], STS, AG, and intraparietal sulcus [IPS]) contained relatively large portions of voxels that showed significant MI or AS values (>30% and >100 voxels within the target ROI). For all target ROIs, there were more MI-only and AS-only voxels than shared voxels (*d* = 2.08 and *d* = 3.30, respectively). The target ROIs showed different patterns of MI-only and AS-only voxels. More MI-only voxels were found in the left STG and STS (left, *d* = 0.79; right, *d* = 0.76) compared with AS-only voxels, whereas more AS-only voxels were found in bilateral IFS (left, *d* = 0.92; right, *d* = 1.26), left PCS (*d* = 1.50), right STG (*d* = 2.38), right AG (*d* = 3.01), and bilateral IPS (left, *d* = 1.54; right, *d* = 3.17) compared with MI-only voxels.

## Discussion

In this current study, participants underwent fMRI experiments and were presented with either unimodal auditory or visual stimuli or with bimodal auditory and visual stimuli; they were later asked to selectively attend to only one modality. The unimodal model, using semantic features, was able to predict the activity in the bilateral inferior frontal, superior temporal, and inferior parietal regions, for both modalities. The involvement of these regions in language processing has been repeatedly suggested in many neuroimaging studies (Price 2010). In contrast, the unimodal models using phonemic features were not able to predict modality-invariant activity. This result is consistent with the results of a previous study, by de Heer et al. (2017), which reported that encoding models using semantic features predicted larger brain regions than those predicted by phonemic models.

Differences in prediction accuracy, between attended and unattended features, can only be explained using the effects of attention as the only differences in the prediction were associated with these extracted features (attended vs. unattended). A behavioral questionnaire administered after each session confirmed that participants showed higher accuracy for understanding the semantic contents of the attended stimuli. This result suggested that semantic information is represented in the brain only when participants pay attention to the target stimuli, which was consistent with the finding that phonemic features produced significant AS values only in very small brain regions.

The brain regions that were well predicted by cross-modal predictions and those regions that were modulated by selective attention demonstrated partial overlap; this agrees with the hypothesis depicted in Fig. 1*A* (center). Anatomical ROI analyses further showed that the overlapping regions were primarily located in perisylvian regions, such as the inferior frontal, superior temporal, and inferior parietal cortices. The MI of semantic representations has been reported previously (Booth et al. 2002; Deniz et al. 2019); however, we show, for the first time, that this representation partially correlates with the effects of selective attention on perisylvian regions.

We also identified cortical voxels showing only MI, as well as those showing only AS. These results suggested heterogeneity among cortical representations of semantic information. A recent study of the cocktail party effect (i.e., the simultaneous presentation of different auditory stimuli) showed that brain activity reflected the semantic information of attended words but not unattended words (Brodbeck et al. 2018). Selective attention may affect some cortical semantic networks, in a modality-specific manner.

Modality-specific regions were primarily identified in the primary auditory cortex, for the auditory modality, and in the primary visual cortex, for the visual modality. These results are consistent with a previous study, which reported that higher-order brain regions were more strongly affected by selective attention than early sensory regions (Regev et al. 2019). Importantly, the modality-specificity values were found to be larger in the early sensory regions, for both semantic and phonemic features, whereas the modality-invariance values were larger in the perisylvian regions, only for semantic features. This model dependency may indicate that the processing of semantic information is more affected by selective attention, whereas phonemic features are primarily processed in early sensory regions and are not fully affected by selective attention.

Additional analyses with sensory regressors showed the reduced MI and AS values only for the phonemic features (Figs 3E,*F* and 5*C*,*D*; Supplementary Figs S8 and S15). In contrast, the sensory regressors did not have much influence on the MS values (Figs 2E,*F* and 3*C*,*D*). The occipital and superior temporal cortices may share linguistic information in a modality-specific manner. This is in line with the previous findings that semantic features are represented in large brain regions, including occipital and superior temporal cortices (Huth et al. 2012; Nishida and Nishimoto 2018).

We used the geometric mean for the definition of MI and AS; however, there are other possible definitions of these values. When we used the arithmetic mean instead of the geometric mean, we obtained similar MI and AS values across the cortex (Supplementary Figs S18–S22). However, we did not use the arithmetic mean in the main analyses because it may include modality-specific components. For example, if one voxel has *D*_S_ = 1 and *D*_T_ = 0 (see Modality Invariance section for the definition of these notations), the arithmetic mean MI = 0.5, whereas the geometric mean MI = 0. Indeed, we found significant MI and AS values in the occipital cortex using the arithmetic mean (Supplementary Figs S18–S21). Thus, we considered that the geometric mean was more appropriate for defining the MI and AS values.

Using an encoding model approach, we compared phonemic and semantic features for their predictabilities of brain activity, which provided detailed information that was not obvious in a previous study that reported increased inter-participant correlations among brain activities during selective attention (Regev et al. 2019). We found that encoding models associated with semantic features were more strongly affected by selective attention than the phoneme-based models, which is consistent with behavioral results showing that the understanding of semantic content was facilitated by selective attention.

Unimodal models using phonemic features have shown modality specificity, not only in the auditory cortex but also in the visual cortex. Although this finding may appear to contradict the idea of a “phoneme,” the MS_T_ values observed in the early visual cortex can be explained by phonemic orthography associated with the Japanese language. Because Japanese largely contains phonograms (i.e., “Hiragana” and “Katakana”), phonemic features may correlate with visually presented Japanese characters.

Encoding models were trained using the stimuli in the unimodal experiment, whereas the bimodal data were used only in the model testing. Adopting this approach allows us to exclude the influence of nontarget modalities from the constructed models, which simplifies the interpretation of the prediction performance (i.e., the cross-modal and attentional effects observed are solely due to the test dataset). However, if we perform bimodal experiments as training, we can visualize how selective attention warps semantic space (e.g., Çukur et al. 2013a, 2013b; Shahdloo et al. 2020). Such detailed analysis would further clarify the types of linguistic content (e.g., noun, verb, and adjective) that are most affected by selective attention.

We used naturalistic, narrative stories and extracted linguistic information from both the auditory and visual stimuli. This approach can be applied to other linguistic features. Although we have only examined two linguistic models, which have both been used in previous studies (de Heer et al. 2017; Nishida and Nishimoto 2018), further applications examining different features, such as syntax, may further increase prediction accuracy and capture more profound representations across modalities. Further modeling approaches are necessary for the comprehensive evaluation of the cortical representation of linguistic information and the effects of selective attention.

## Authors’ Contribution

T.N., H.Q.Y., and S.N. designed the study. T.N. and H.Q.Y. collected and analyzed the data. T.N., H.Q.Y., and S.N. wrote the manuscript.

## Funding

MEXT/JSPS KAKENHI (grant numbers JP20K07718, JP20H05023 in #4903 (Evolinguistics) to T.N., JP20J12959 to H.Q.Y., and JP15H05311 to S.N.), as well as JST (CREST JPMJCR18A5 and ERATO JPMJER1801 to S.N.), for the partial financial support of this study. The funders had no role in the study design, data collection, and analysis, decision to publish, or preparation of the manuscript.

## Notes

*Conflict of Interest*: The authors declare no competing interests.

## Supplementary Material

SI_bhab125
