# Supplementary material for: Convergence of Modality Invariance and Attention Selectivity in the Cortical Semantic Circuit
Source: Cereb Cortex. 2021 May 17;31(10):4825–39. doi: 10.1093/cercor/bhab125 (PMC8408468; doi:10.1093/cercor/bhab125)
Supplement: SI_bhab125 [file si_bhab125.pdf]

Convergence of attention selectivity and modality invariance in the cortical semantic circuit

Tomoya Nakai, Hiroto Q. Yamaguchi, Shinji Nishimoto

Supplementary Information

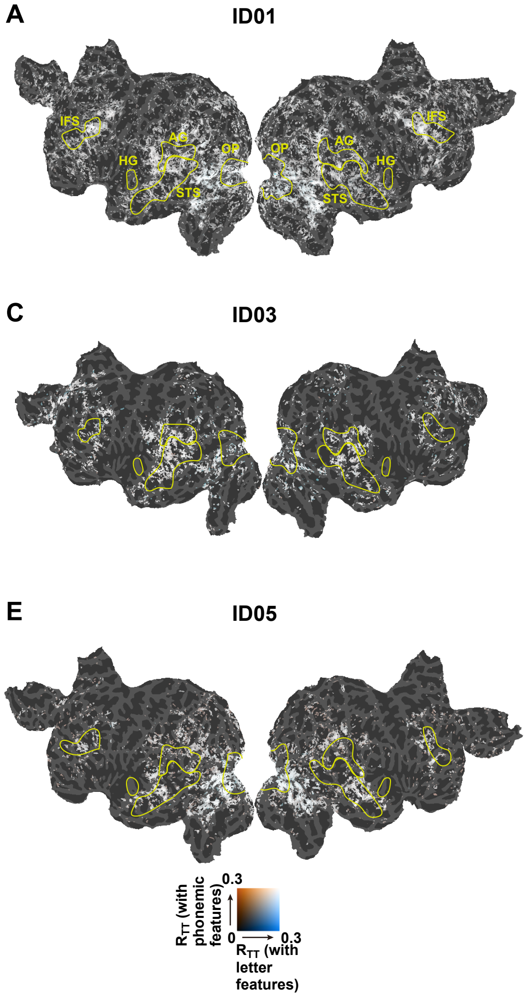

Figure S1. Overlap of prediction accuracy maps using letter and phonemic features. The intra-modal prediction accuracies of the Text-only model (denoted as R_{TT}) were calculated using letter (blue) and phonemic (red) features, and mapped onto the cortical surface of participants ID01–ID06. Only significant regions ($p < 0.05$, FDR corrected) are shown. Anatomical regions of interest are marked by yellow lines. IFS, inferior frontal sulcus; STS, superior temporal sulcus; AG, angular gyrus; OP, occipital pole; HG, Heschl's gyrus.

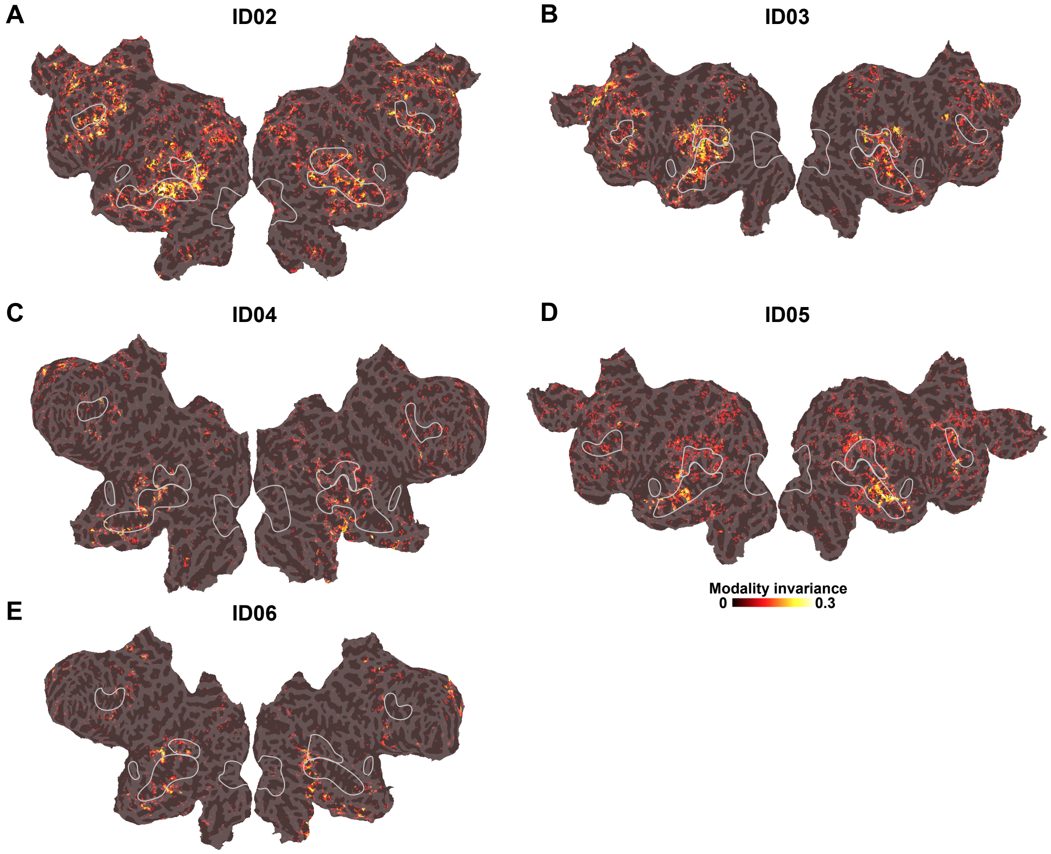

Figure S2. Modality invariance using semantic features. Modality invariance was calculated using semantic features and mapped onto the cortical surface of participants ID02–ID06. Only significant regions ($p < 0.05$, FDR corrected) are shown.

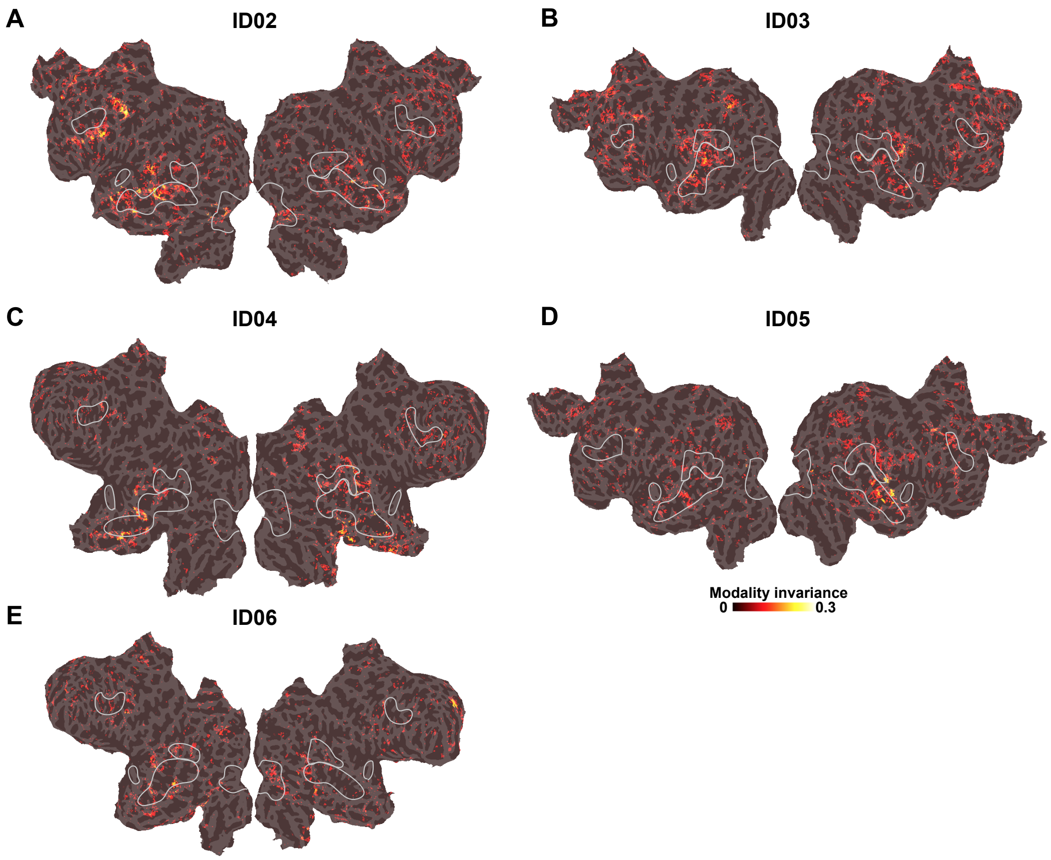

Figure S3. Modality invariance using phonemic features. Modality invariance was calculated using phonemic features and mapped onto the cortical surface of participants ID02–ID06. Only significant regions ($p < 0.05$, FDR corrected) are shown.

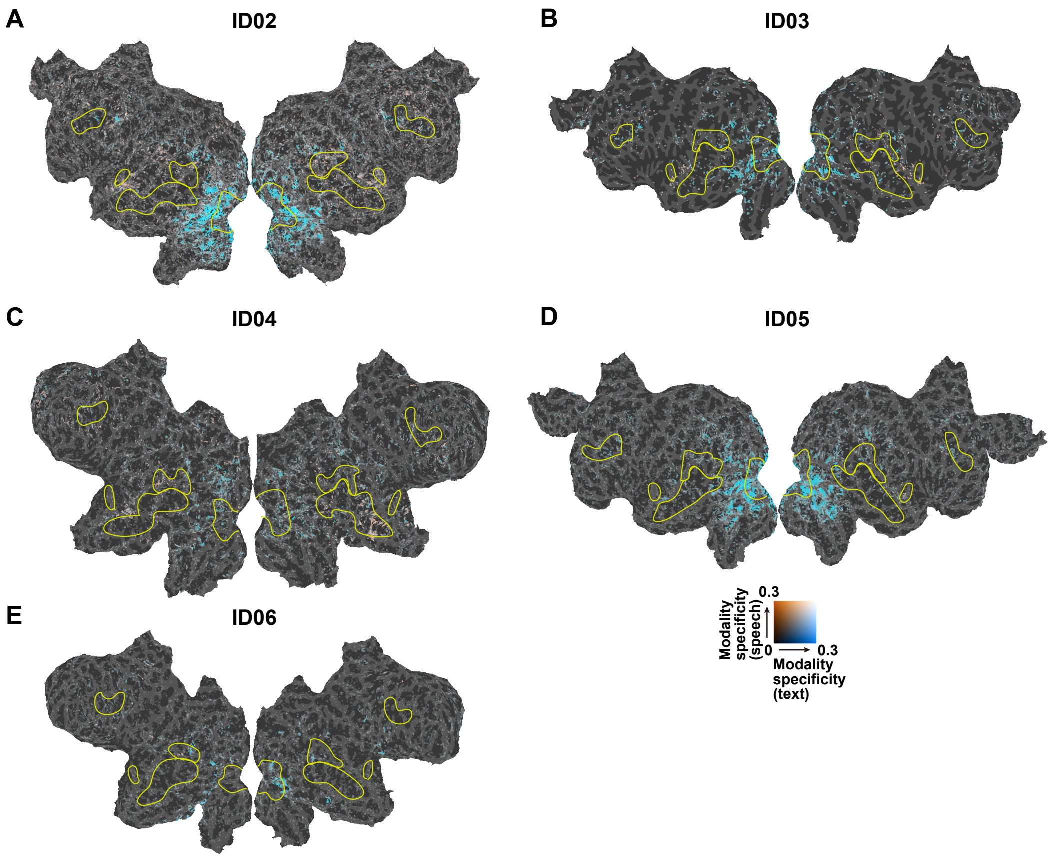

Figure S4 Modality specificity using semantic features. Modality specificity in text (blue), and modality specificity in speech (red) were calculated using semantic features and mapped onto the cortical surface of participants ID02–ID06. Only significant regions ($p < 0.05$, FDR corrected) are shown in each color.

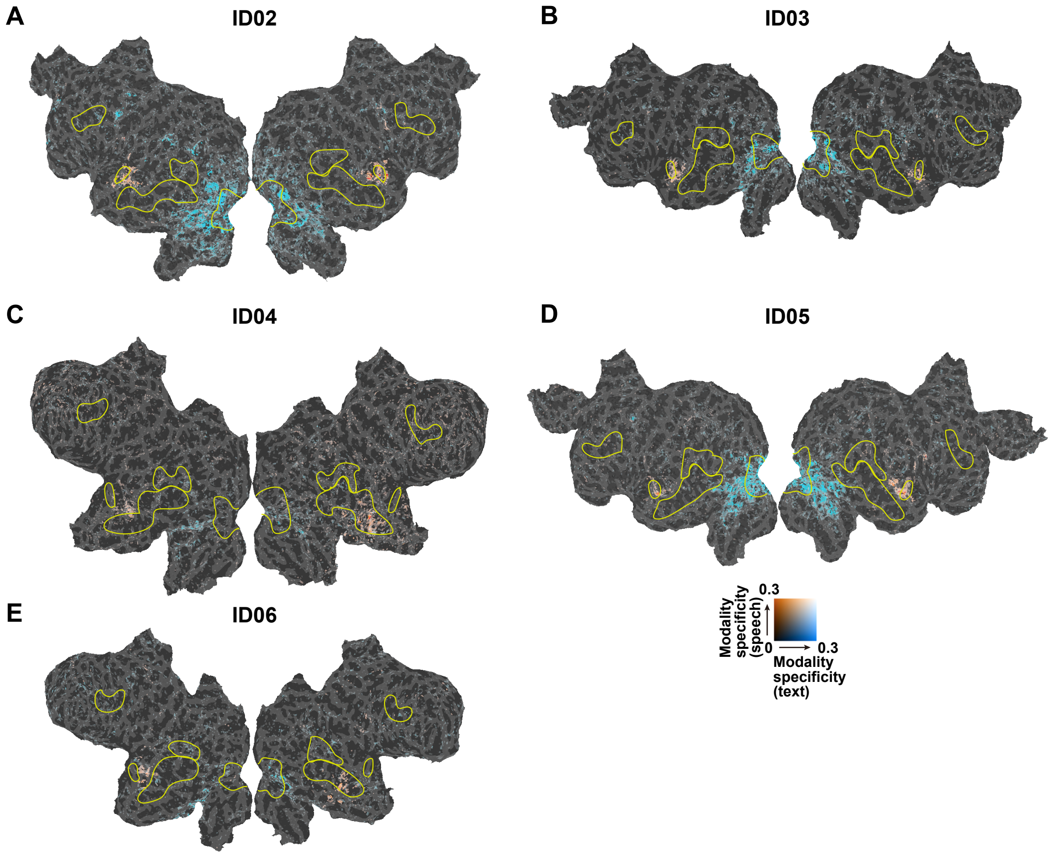

Figure S5. Modality specificity using semantic features. Modality specificity in text (blue), and modality specificity in speech (red) were calculated using phonemic features and mapped onto the cortical surface of participants ID02–ID06. Only significant regions ($p < 0.05$, FDR corrected) are shown in each color.

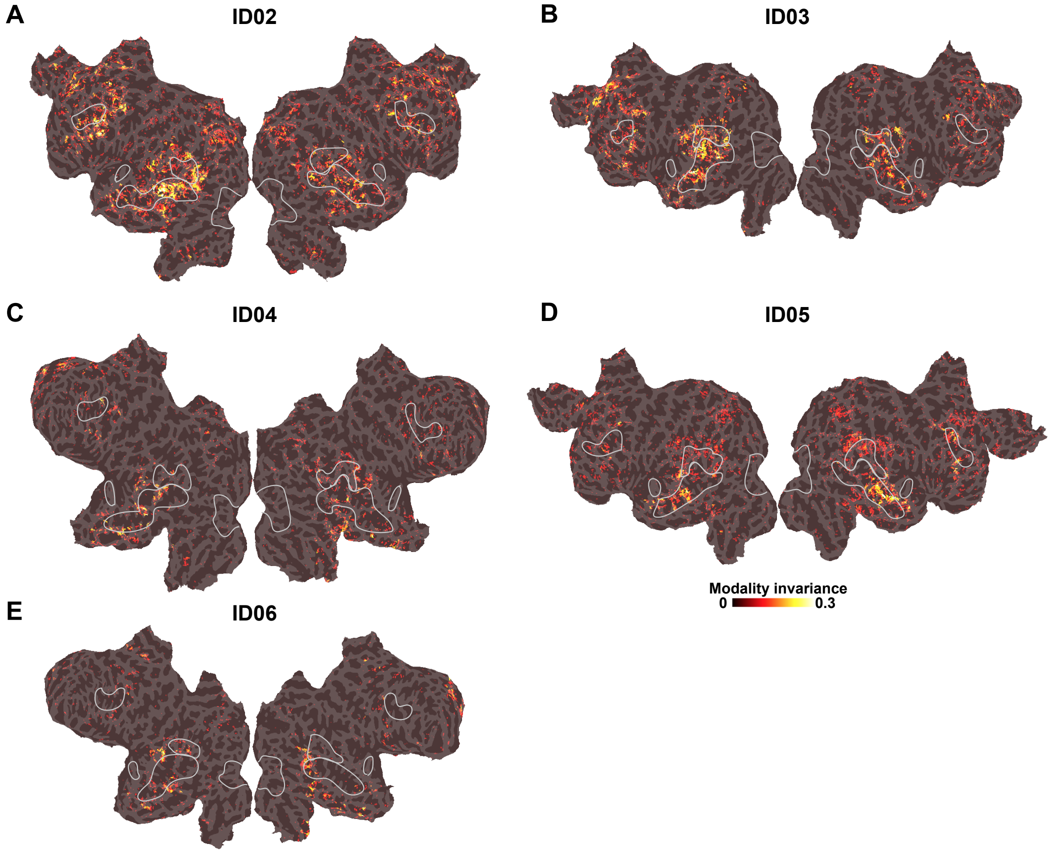

Figure S6. Modality invariance using semantic features and regressing out sensory components.

Modality invariance was calculated using semantic features and regressing out sensory components and are mapped onto the cortical surface of participants ID02–ID06. Only significant regions ($p < 0.05$, FDR corrected) are shown.

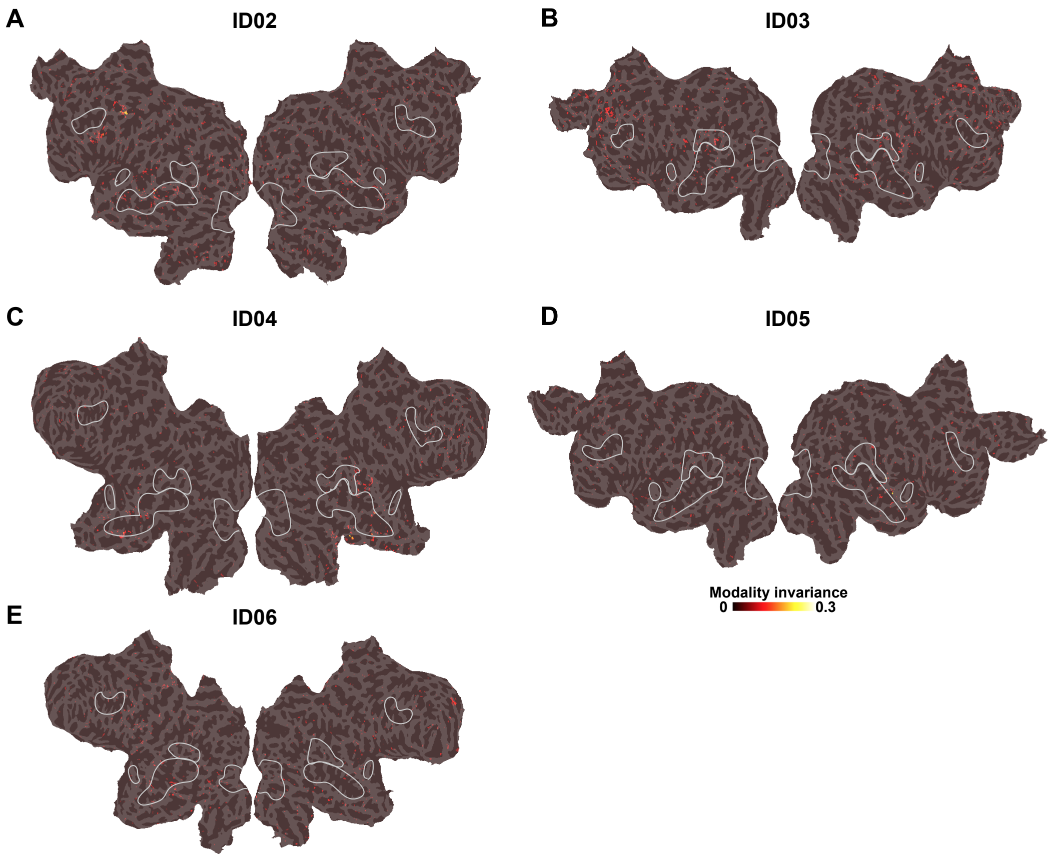

Figure S7. Modality invariance using phonemic features and regressing out sensory components.

Modality invariance was calculated using phonemic features and regressing out sensory components and are mapped onto the cortical surface of participants ID02–ID06. Only significant regions ($p < 0.05$, FDR corrected) are shown.

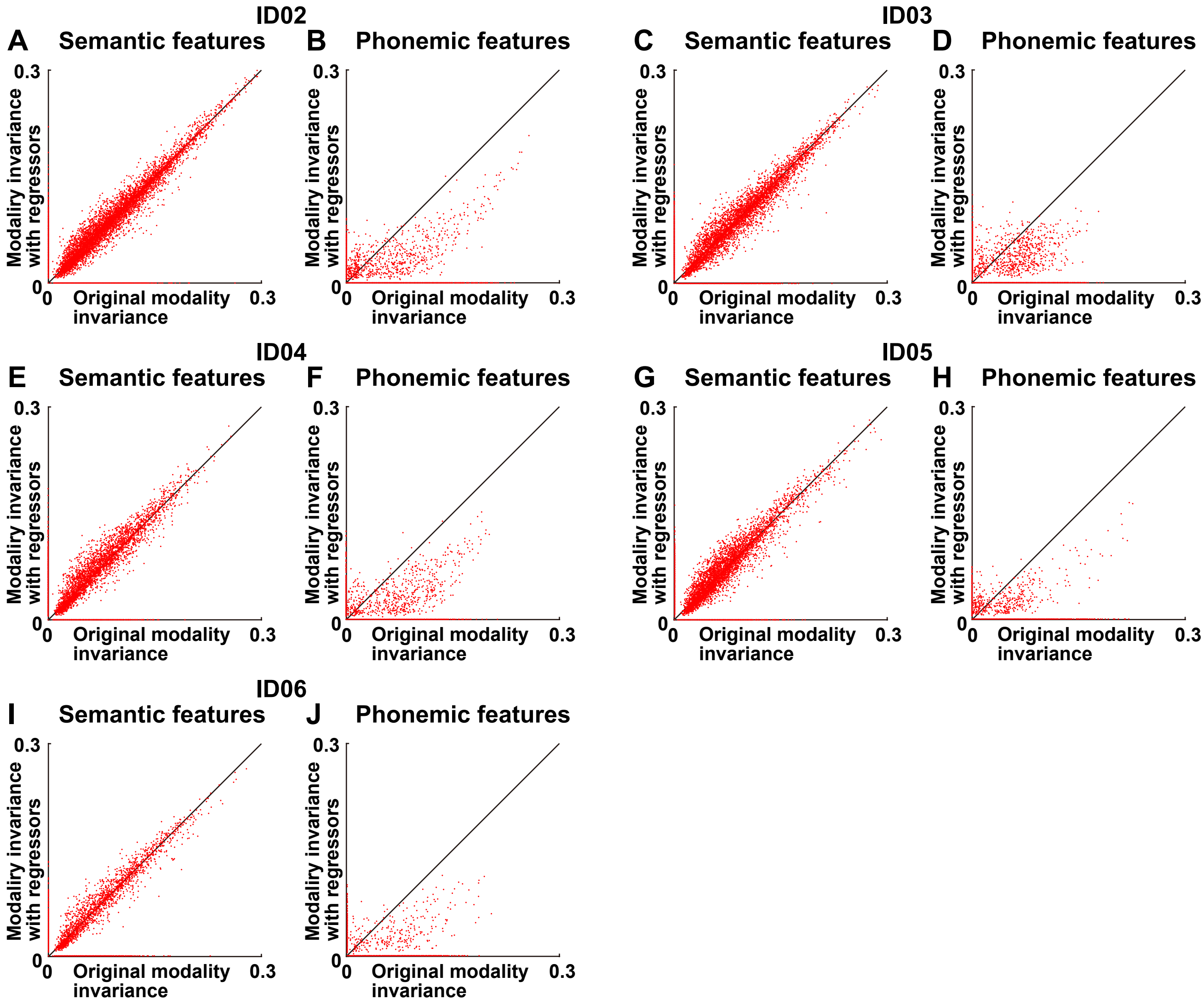

Figure S8. Effect of sensory regressors on modality invariance. Scatter plots show the original modality invariance, and modality invariance with sensory regressors, for both semantic and phonemic features, plotted for participants ID02–ID06. Motion energy and modulation transfer function features were used as regressors.

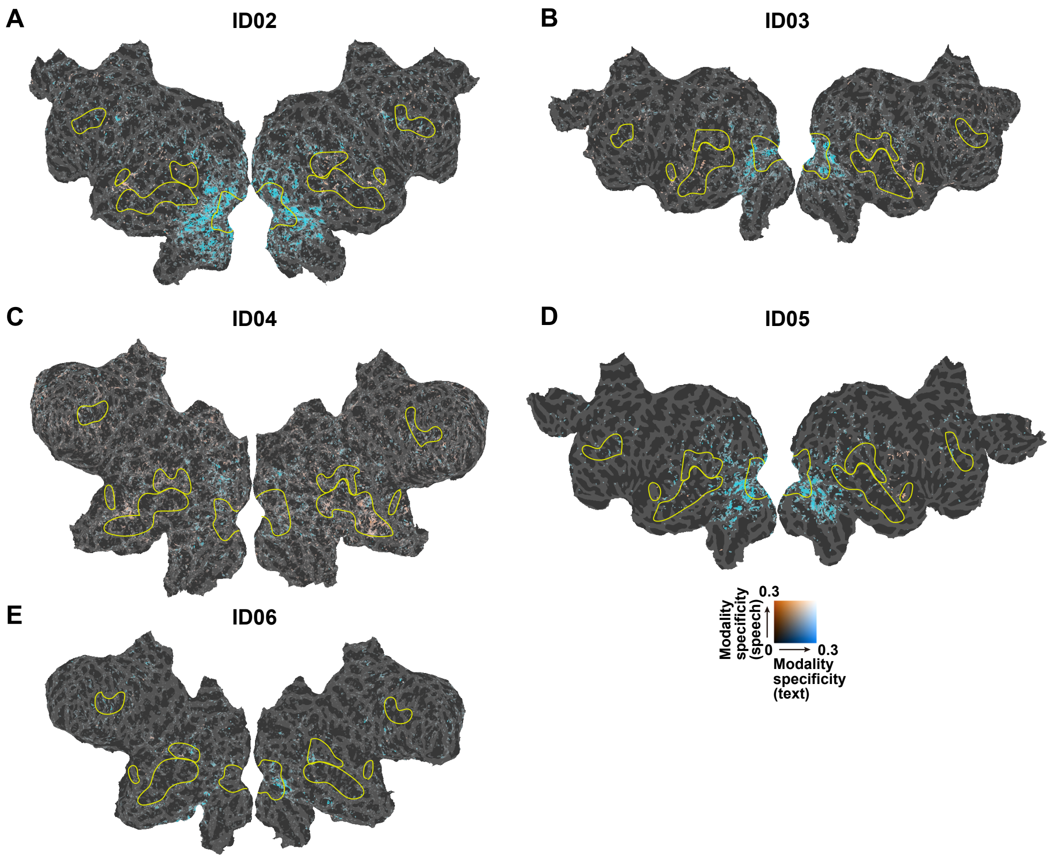

Figure S9. Modality specificity using semantic features and regressing out sensory components.

Modality specificity in text (blue) and modality specificity in speech (red) were calculated using semantic features and regressing out sensory components and are mapped onto the cortical surface of participants ID02–ID06. Only significant regions ($p < 0.05$, FDR corrected) are shown in each color.

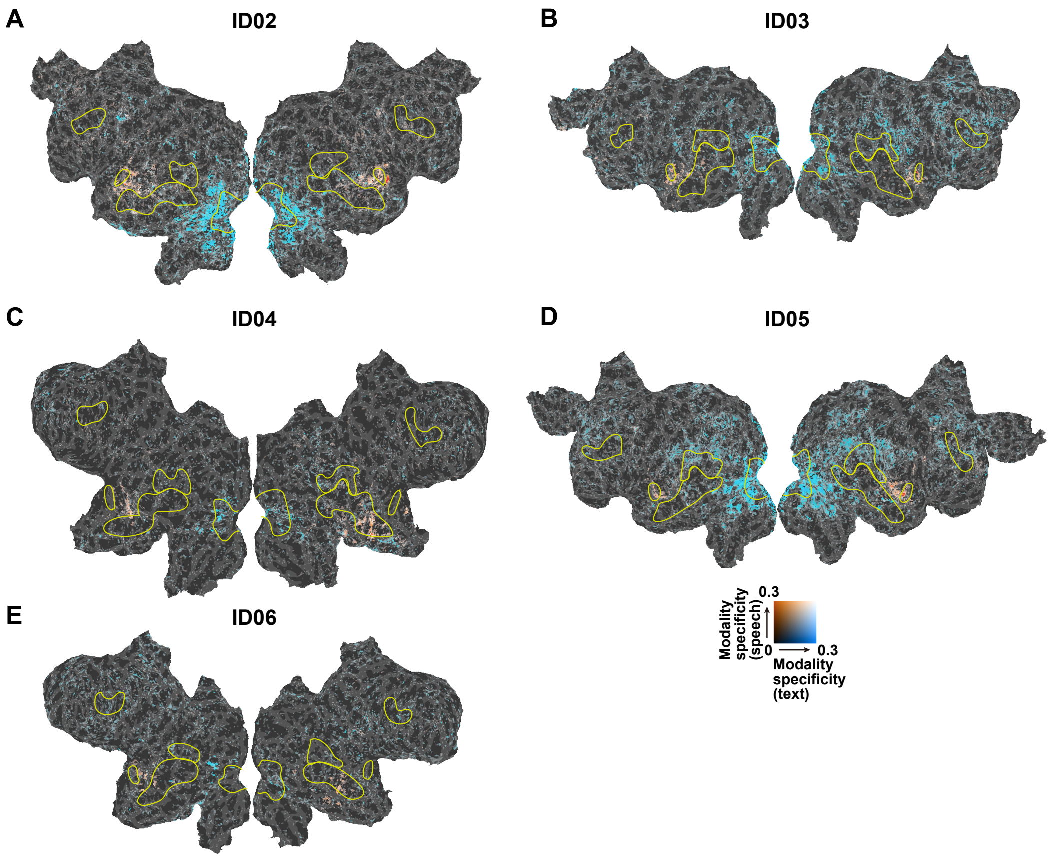

Figure S10. Modality specificity using phonemic features and regressing out sensory components. Modality specificity in text (blue) and modality specificity in speech (red) were calculated using phonemic features and regressing out sensory components and are mapped onto the cortical surface of participants ID02–ID06. Only significant regions ($p < 0.05$, FDR corrected) are shown in each color.

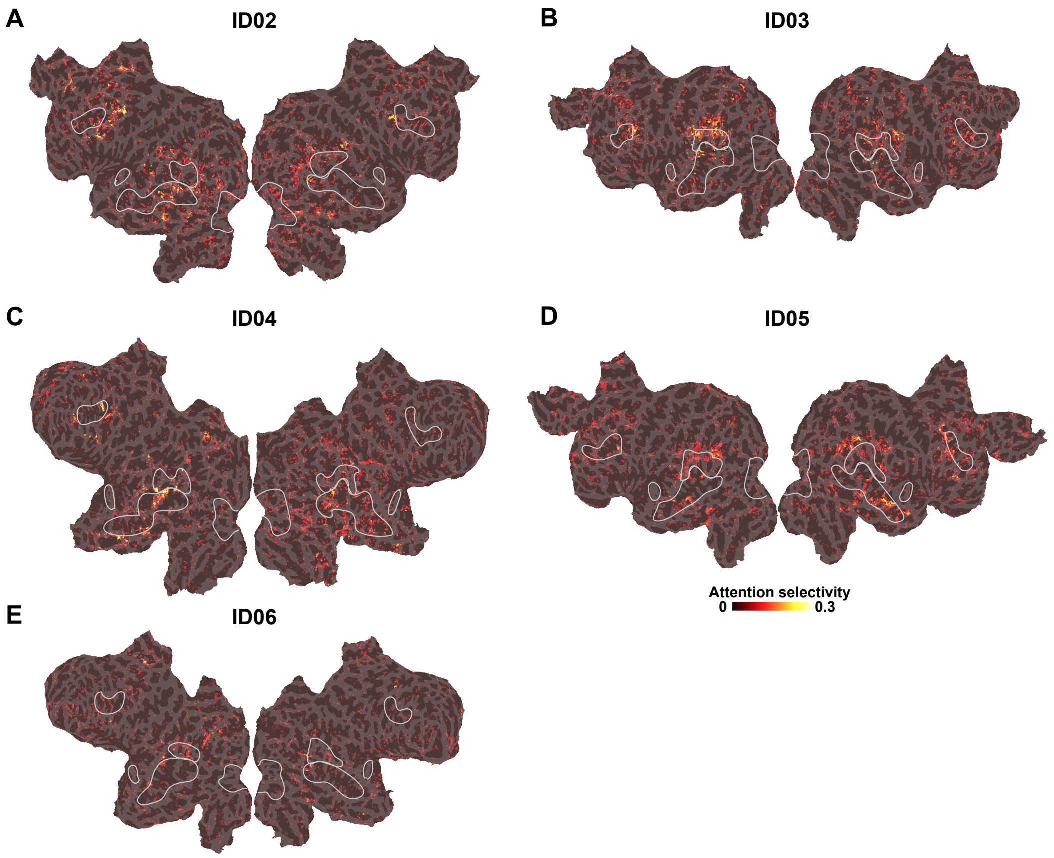

Figure S11. Attention selectivity using semantic features. Attention selectivity was calculated using semantic features and mapped onto the cortical surface of participants ID02–ID06. Only significant regions ($p < 0.05$, FDR corrected) are shown.

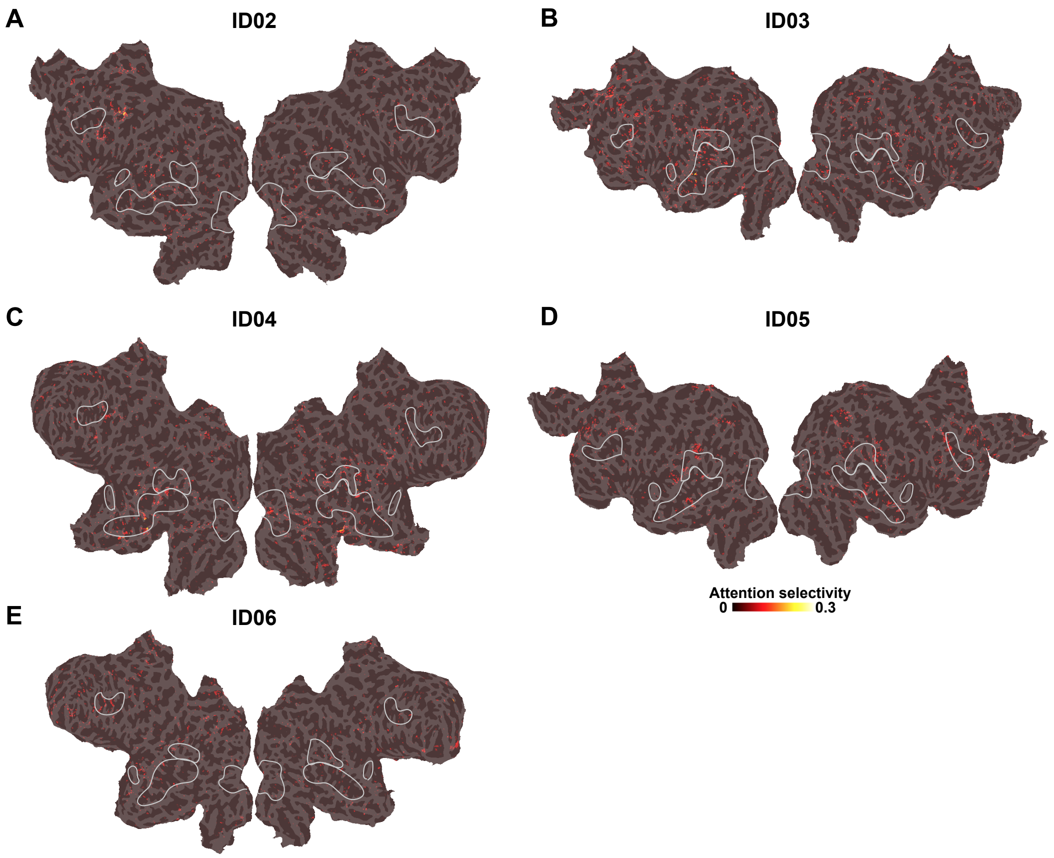

Figure S12. Attention selectivity using phonemic features. Attention selectivity was calculated using phonemic features, mapped onto the cortical surface of participants ID02–ID06. Only significant regions ($p < 0.05$, FDR corrected) are shown.

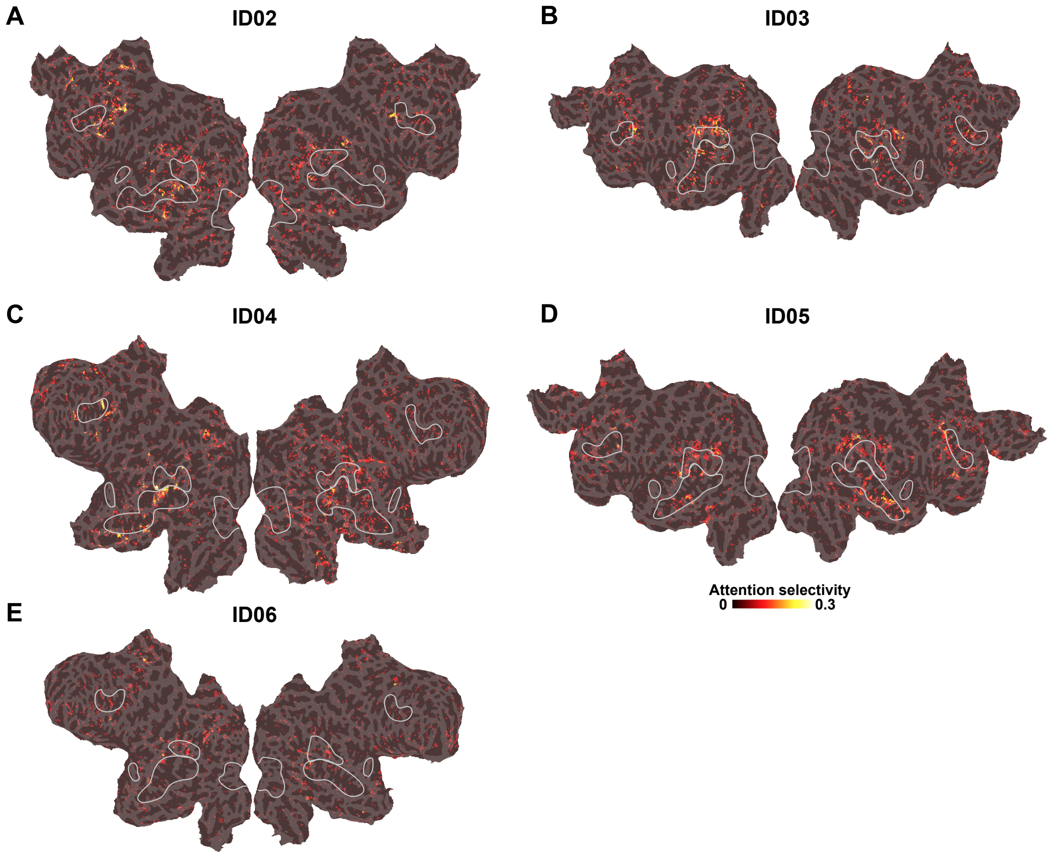

Figure S13. Attention selectivity using semantic features and regressing out sensory components. Attention selectivity was calculated using semantic features and regressing out sensory components and are mapped onto the cortical surface of participants ID02–ID06. Only significant regions ($p < 0.05$, FDR corrected) are shown.

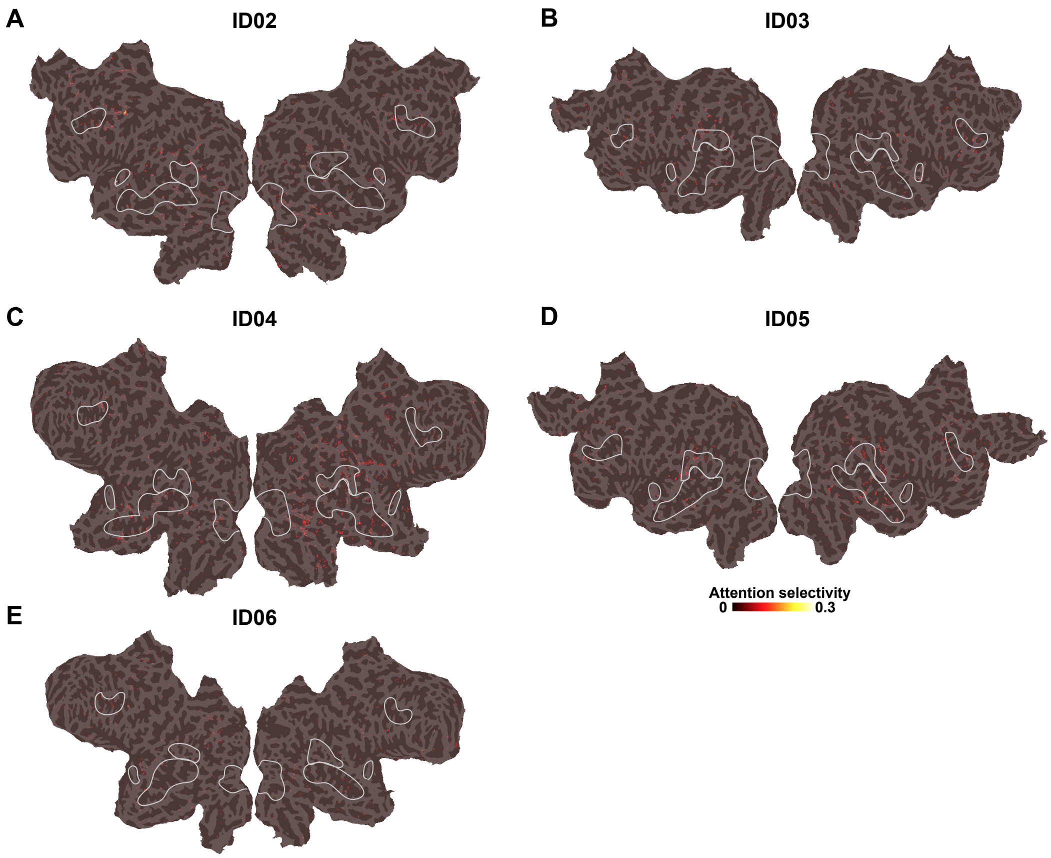

Figure S14. Attention selectivity using phonemic features and regressing out sensory components. Attention selectivity was calculated using phonemic features and regressing out sensory components and are mapped onto the cortical surface of participants ID02–ID06. Only significant regions ($p < 0.05$, FDR corrected) are shown.

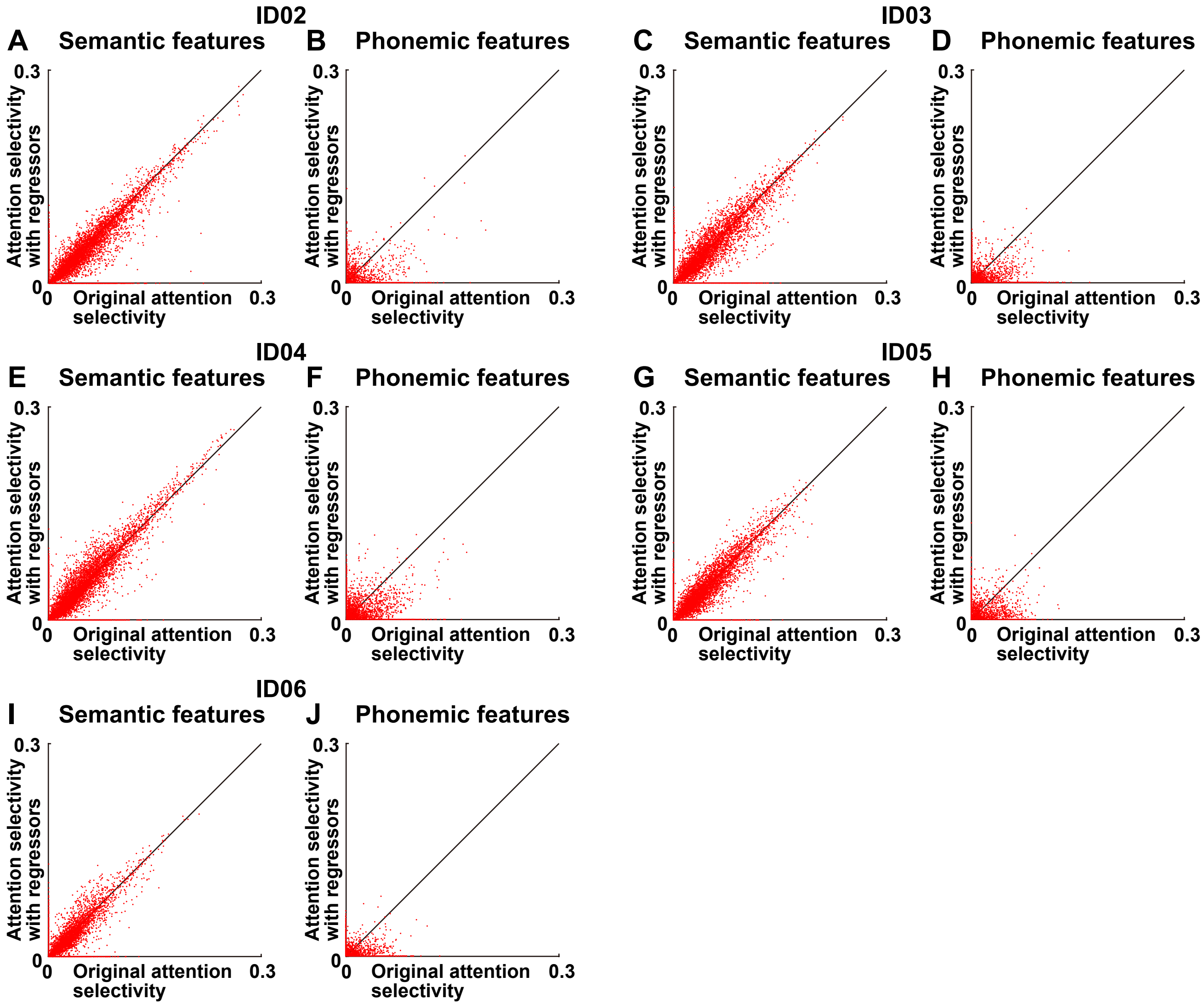

Figure S15. Effect of sensory regressors on attention selectivity. Scatter plots show the original attention selectivity, and attention selectivity with sensory regressors, for both semantic and phonemic features, plotted for participants ID02–ID06. Motion energy and modulation transfer function features were used as regressors.

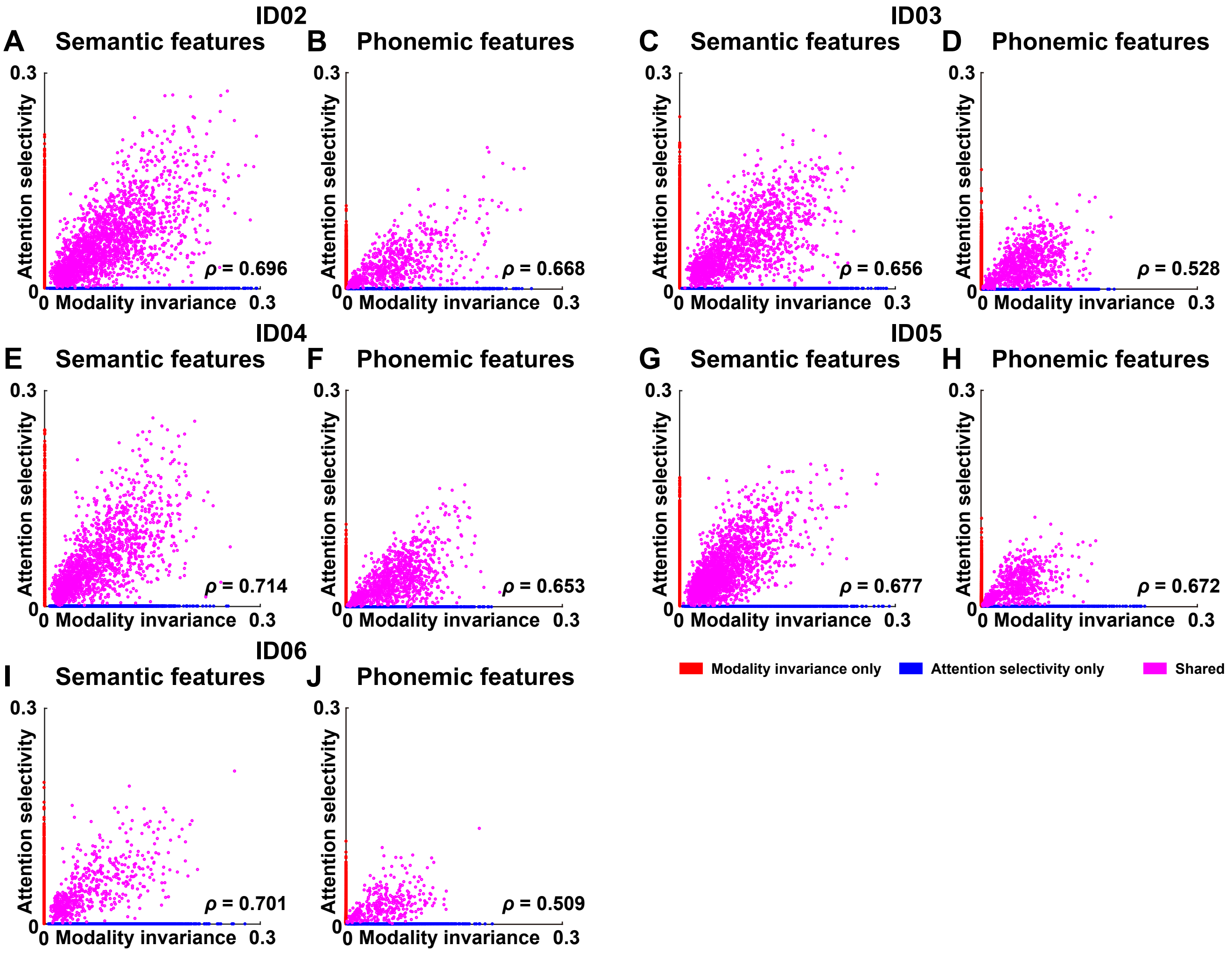

Figure S16. Scatter plots of modality invariance and attention selectivity. Scatter plots are shown for modality invariance and attention selectivity using semantic features and phonemic features, extracted from the cortical voxels of participants ID02–ID06. For each plot, a Spearman's correlation coefficient (ρ) is displayed.

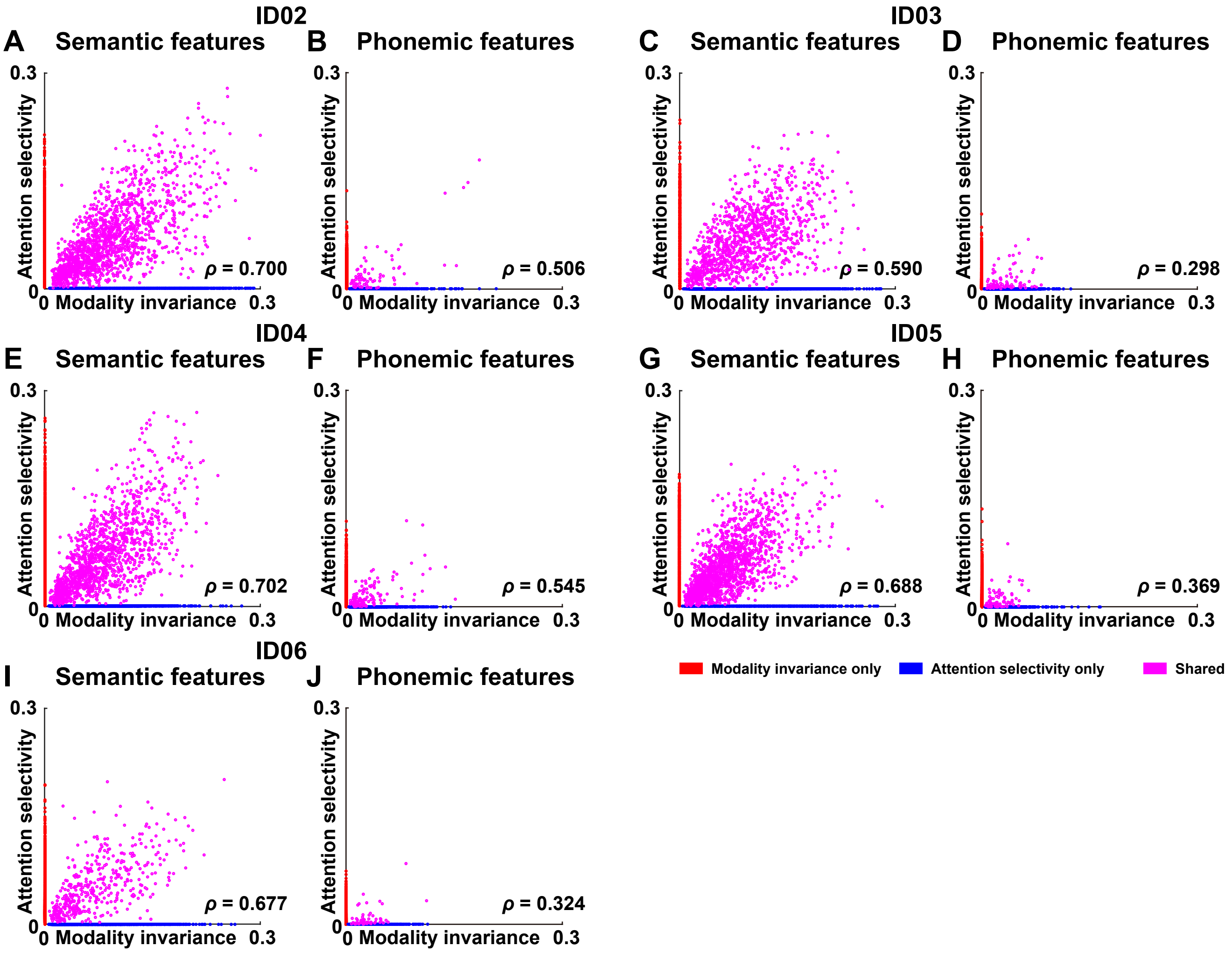

Figure S17. Scatter plots of modality invariance and attention selectivity with sensory components regressed out. Scatter plots show modality invariance and attention selectivity using semantic and phonemic features, with sensory components regressed out and extracted from the cortical voxels of participants ID02–ID06. Spearman's correlation coefficients (ρ) are displayed for each plot.

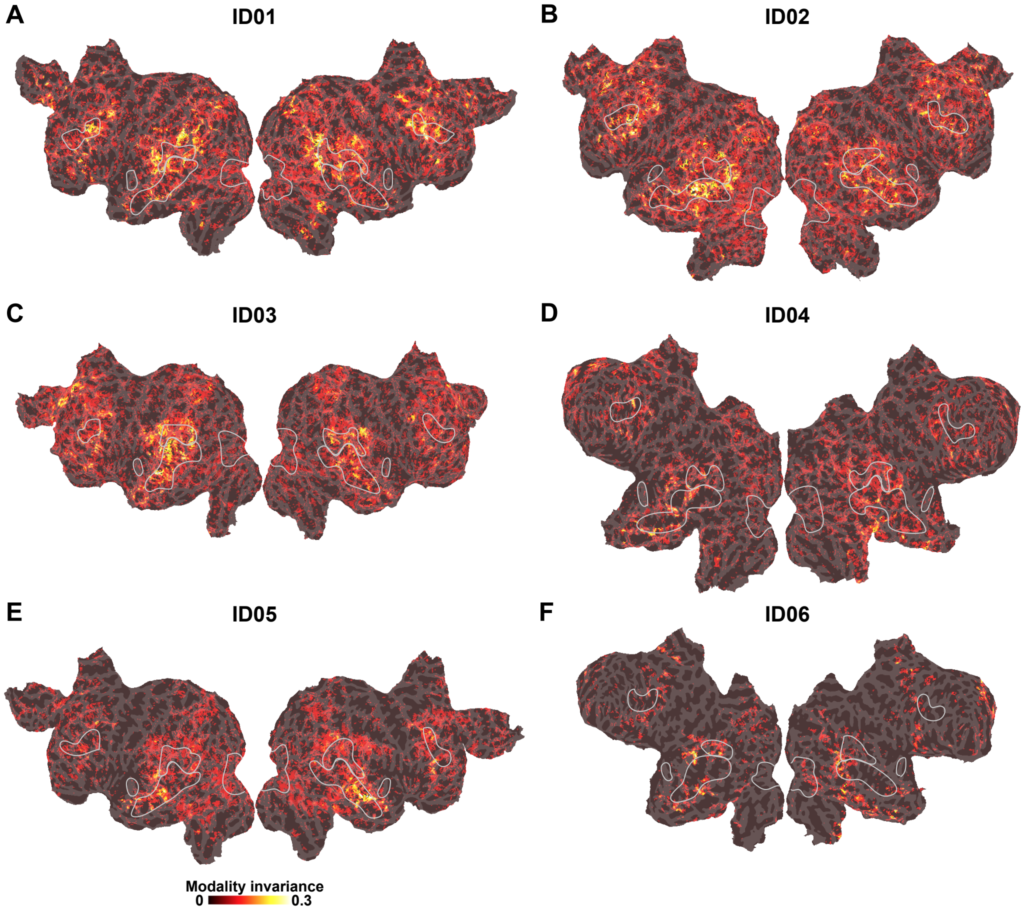

Figure S18. Modality invariance with the arithmetic mean using semantic features. Modality invariance was calculated using the semantic features, using the arithmetic mean instead of the geometric mean, and mapped onto the cortical surface of participants ID01–ID06. Only significant regions (p < 0.05, FDR corrected) are shown.

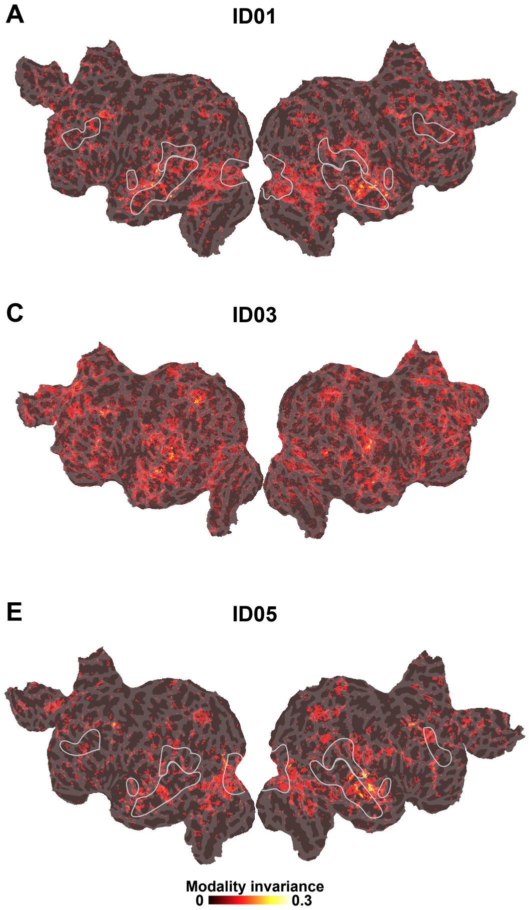

Figure S19. Modality invariance with the arithmetic mean using phonemic features. Modality invariance was calculated using the phonemic features, using the arithmetic mean instead of the geometric mean, and mapped onto the cortical surface of participants ID01–ID06. Only significant regions ($p < 0.05$, FDR corrected) are shown.

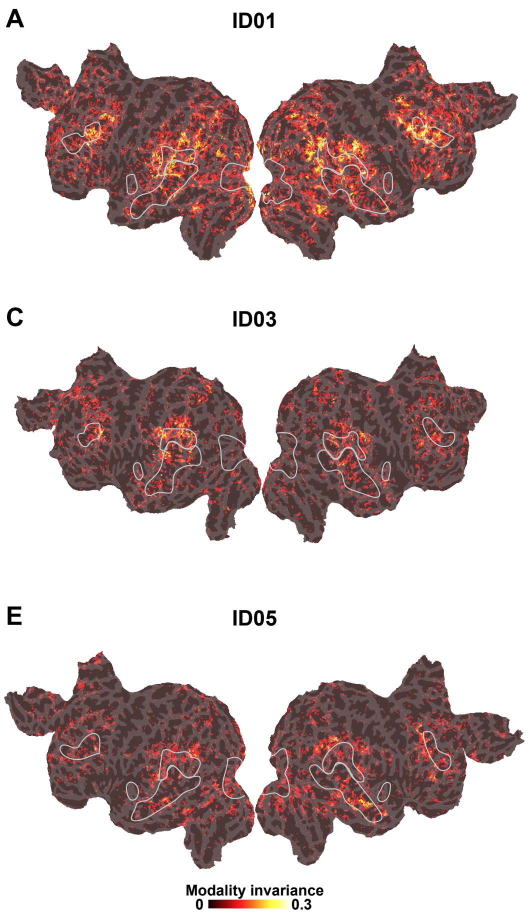

Figure S20. Attention selectivity with arithmetic mean using semantic features. Attention selectivity was calculated using semantic features, using arithmetic mean instead of geometric mean, and mapped onto the cortical surface of participant ID01–ID06. Only significant regions ($p < 0.05$, FDR corrected) are shown.

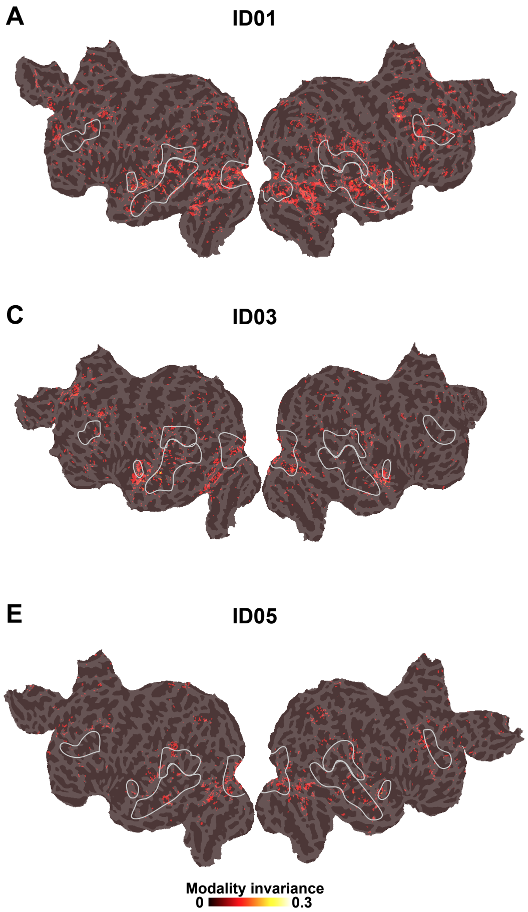

Figure S21. Attention selectivity with arithmetic mean using phonemic features. Attention selectivity was calculated using phonemic features, using arithmetic mean instead of geometric mean, and mapped onto the cortical surface of participant ID01–ID06. Only significant regions ($p < 0.05$, FDR corrected) are shown.

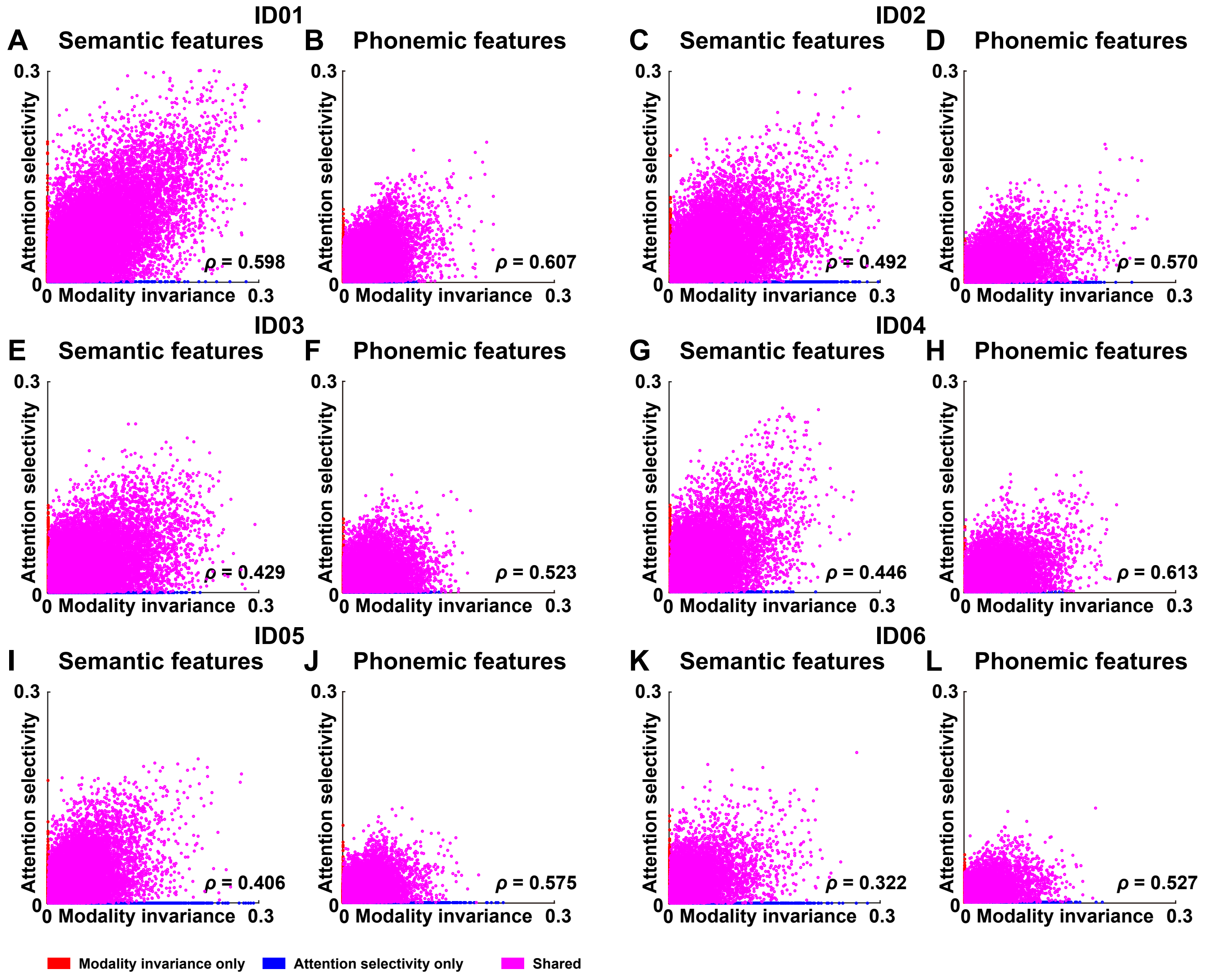

Figure S22. Scatter plots of modality invariance and attention selectivity using the arithmetic mean. Scatter plot are shown for modality invariance and attention selectivity using semantic features and phonemic features, using the arithmetic mean instead of the geometric mean, and extracted from the cortical voxels of participants ID01–ID06. For each plot, a Spearman's correlation coefficients (ρ) are displayed for each plot.
